# Enzyme intermediates captured “on the fly” by mix-and-inject serial crystallography

**DOI:** 10.1186/s12915-018-0524-5

**Published:** 2018-05-31

**Authors:** Jose L. Olmos, Suraj Pandey, Jose M. Martin-Garcia, George Calvey, Andrea Katz, Juraj Knoska, Christopher Kupitz, Mark S. Hunter, Mengning Liang, Dominik Oberthuer, Oleksandr Yefanov, Max Wiedorn, Michael Heyman, Mark Holl, Kanupriya Pande, Anton Barty, Mitchell D. Miller, Stephan Stern, Shatabdi Roy-Chowdhury, Jesse Coe, Nirupa Nagaratnam, James Zook, Jacob Verburgt, Tyler Norwood, Ishwor Poudyal, David Xu, Jason Koglin, Matthew H. Seaberg, Yun Zhao, Saša Bajt, Thomas Grant, Valerio Mariani, Garrett Nelson, Ganesh Subramanian, Euiyoung Bae, Raimund Fromme, Russell Fung, Peter Schwander, Matthias Frank, Thomas A. White, Uwe Weierstall, Nadia Zatsepin, John Spence, Petra Fromme, Henry N. Chapman, Lois Pollack, Lee Tremblay, Abbas Ourmazd, George N. Phillips, Marius Schmidt

**Affiliations:** 1 Department of BioSciences, Rice University, 6100 Main Street, Houston, TX 77005 USA; 2 Physics Department, University of Wisconsin-Milwaukee, 3135 N. Maryland Ave, Milwaukee, WI 53211 USA; 3 School of Molecular Sciences and Biodesign Center for Applied Structural Discovery, Arizona State University, Tempe, AZ 85287-1604 USA; 4 School of Applied and Engineering Physics, Cornell University, 254 Clark Hall, Ithaca, NY, 14853 USA; 5 Center for Free-Electron Laser Science, DESY, Notkestrasse 85, 22607 Hamburg, Germany; 6 University of Hamburg, Luruper Chaussee 149, 22761 Hamburg, Germany; 7 Linac Coherent Light Source, Stanford Linear Accelerator Center (SLAC) National, Accelerator Laboratory, 2575 Sand Hill Road, Menlo Park, CA, 94025 USA; 8 Max Planck Institut fuer Biochemie, Am Klopferspitz 18, 82152 Planegg, Germany; 9 Lawrence Berkeley National Lab, 1 Cyclotron Road, Berkeley, CA 94720 USA; 10 Milwaukee School of Engineering, Milwaukee, WI 53202-3109 USA; 11 Photon Science, DESY, Notkestrasse 85, 22607 Hamburg, Germany; 12 University of New York Buffalo, Hauptman-Woodward Institute, 700 Ellicott St, Buffalo, NY 14203 USA; 13 Department of Physics, Arizona State University, Tempe, AZ 85287 USA; 14 Department of Agricultural Biotechnology, Seoul National University, Seoul, 08826 Korea; 15 Lawrence Livermore National Laboratory, Livermore, CA 94550 USA; 16 Centre for Ultrafast Imaging, Luruper Chaussee 149, 22761 Hamburg, Germany; 17 4Marbles Inc., 1900 Belvedere Pl, Westfield, IN 46074 USA; 18 GlaxoSmithKline, Gunnels Wood Road, Stevenage, SG1 2NY UK

## Abstract

**Background:**

Ever since the first atomic structure of an enzyme was solved, the discovery of the mechanism and dynamics of reactions catalyzed by biomolecules has been the key goal for the understanding of the molecular processes that drive life on earth. Despite a large number of successful methods for trapping reaction intermediates, the direct observation of an ongoing reaction has been possible only in rare and exceptional cases.

**Results:**

Here, we demonstrate a general method for capturing enzyme catalysis “in action” by *mix-and-inject serial crystallography (MISC)*. Specifically, we follow the catalytic reaction of the *Mycobacterium tuberculosis* β-lactamase with the third-generation antibiotic ceftriaxone by time-resolved serial femtosecond crystallography. The results reveal, in near atomic detail, antibiotic cleavage and inactivation from 30 ms to 2 s.

**Conclusions:**

MISC is a versatile and generally applicable method to investigate reactions of biological macromolecules, some of which are of immense biological significance and might be, in addition, important targets for structure-based drug design. With megahertz X-ray pulse rates expected at the Linac Coherent Light Source II and the European X-ray free-electron laser, multiple, finely spaced time delays can be collected rapidly, allowing a comprehensive description of biomolecular reactions in terms of structure and kinetics from the same set of X-ray data.

**Electronic supplementary material:**

The online version of this article (10.1186/s12915-018-0524-5) contains supplementary material, which is available to authorized users.

## Background

Observing the catalytic action of a biomolecule in atomic detail has been the dream of structural biologists since the first structure of an enzyme was solved [1, 2]. By exploiting X-ray radiation from powerful synchrotron sources, time-resolved crystallographic methods were developed [3] with the goal of achieving a complete description of a reaction in real time [4, 5]. However, X-ray damage and the need for large single crystals made time-resolved crystallography very challenging. The advent of X-ray free-electron lasers (XFELs) has enabled time-resolved serial femtosecond (fs) crystallography (SFX), where X-ray damage is outrun by ultrashort fs X-ray pulses [6, 7]. This approach has made it possible to follow and describe cyclic and non-cyclic reactions triggered by light. Examples include pioneering studies on the photoactive yellow protein [8, 9], myoglobin [10], bacteriorhodopsin [11], photoswitchable fluorescent proteins [12, 13], and photosystem II [14–17]. However, structural investigations on one-pathway enzymatic reactions present additional difficulties, because diffusion of substrate(s) and products in and out of the crystals limit the accessible reaction times. Standard crystallography can be used to track reaction intermediates of slow reactions by flash cooling [18–20], but the method is then unable to reveal enzymatic reactions at room temperature in real time. The problem is to start a reaction in large-sized crystals. Initiation by diffusion is far slower in these crystals than the typical millisecond turnover times of enzymes. It was proposed that one can trigger enzymatic reactions by light by soaking inactive (caged) substrates [21] into the crystals, which then can be activated by a laser pulse. The first proof of concept for time-resolved Laue crystallography triggered by a caged substrate was achieved in 1990 [22]. While this method has great potential, its application has so far been limited due to significant experimental challenges. Only a few time-resolved experiments have been reported where highly reactive, caged substrates are readily available [18, 22, 23], or the reactions are slow and allow the use of more conventional methods [24, 25]. It is therefore highly desirable to develop new methods that open the field of time-resolved crystallography to the study of biomolecular reactions at room temperature with the native enzyme and its natural substrate(s).

Structural studies at XFELs offer the possibility of a breakthrough. The XFEL intensity is high enough to generate a diffraction pattern from an exposure to a single fs X-ray pulse even from micrometer- and submicrometer-sized crystals. These tiny crystals allow for fast (sub-millisecond to millisecond) diffusion times, which are not rate-limiting for many enzymatic reactions [26–32]. The microcrystals are mixed “on the fly” and injected into the XFEL beam, a method we call *mix-and-inject serial crystallography (MISC)* [28, 30]. In MISC, crystals react with their native substrate(s) at ambient temperature until they are probed by a single X-ray pulse that destroys them but not before a diffraction pattern has been recorded. The pulses are short enough to essentially outrun radiation damage by means of the “diffraction-before-destruction” principle [33–35]. Optimized injectors have been recently developed [36, 37] for MISC experiments with the potential to provide submillisecond time resolution [38]. The microcrystals may tolerate even larger conformational changes leading to unit cell or even space group changes [14, 31].

Here, we apply MISC to the study of a very important public health problem: bacterial antibiotic resistance. Specifically, we have obtained time-resolved crystallographic data on the binding and cleavage of the third-generation antibiotic ceftriaxone (CEF) in microcrystals of the enzyme β-lactamase from *Mycobacterium tuberculosis* (BlaC). Previous studies introduced mutations into BlaC by exchanging catalytically important residues to slow down (or stop) the reaction to the extent that the binding of numerous antibiotics to BlaC could be studied [39]. In our experiments, however, carried out at the Linac Coherent Light Source (LCLS), microcrystals of unmodified BlaC are mixed with CEF on the fly, and the cleavage and thereby inactivation of the antibiotics by the wild-type β-lactamase is followed at runtime. BlaC is a broad-spectrum β-lactamase which confers resistance to all classes of β-lactam antibiotics in tuberculosis [19, 40]. BlaC chemistry has rendered the frontline arsenal of antibacterial agents ineffective against this deadly disease, creating a global public health crisis.

**Fig. 1:**
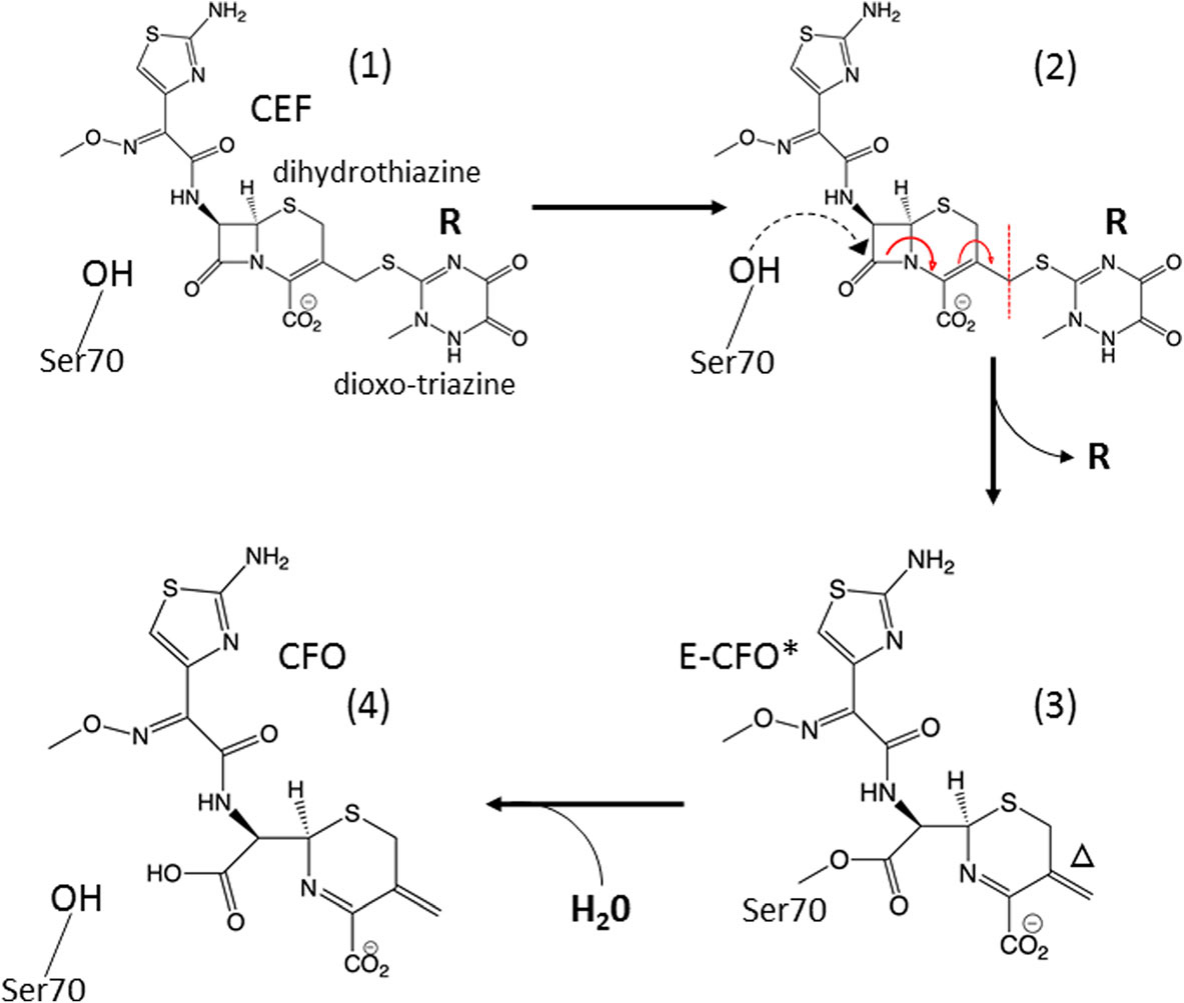
Reaction of β-lactamase with ceftriaxone (*CEF*). (**1**) Formation of the enzyme substrate complex by non-covalently binding CEF. (**2**) Nucleophilic attack of the active site residue Ser-70 results in rearrangement of double bonds and ultimately leads to the opening of the β-lactam ring (*blue arrow* points to the bond to be cleaved) and the detachment of the leaving group (*R*). **(3)** Covalent bond formation between Ser-70 and a shortened species (E-CFO*). Note the double bond *∆*. The double bond may react with water to form an alcohol (OH). Evidence for all four intermediate species is found in our experiments. (**4**) Species (**3**) is further hydrolyzed from Ser-70 and leaves the enzyme as product

Beginning with the famous discovery of penicillin, β-lactam antibiotics were widely used to eliminate deadly bacterial infectious diseases [41]. More compounds with diverse chemical composition have been found through the years [42], the most prominent of them most likely the cephalosporins. The chemical structure of CEF is shown in Fig. 1. Unlike the penicillins, which feature a 5-membered thiazolidine ring, in the cephalosporins a 6-membered dihydrothiazine ring is fused to the β-lactam ring. However, rampant resistance against these antibiotics was observed shortly after their widespread use [41]. β-lactamases open the β-lactam ring, thereby rendering the antibiotic inactive. BlaC from *M. tuberculosis*, an Ambler class A β-lactamase [43], uses a conserved serine to attack the β-lactam ring (Fig. 1, blue arrow), thus inactivating the antibiotics. Because of the medical challenge that BlaC causes for the fight against infectious diseases, the process of catalysis has been studied by conventional biochemical methods in detail, leading to the hypothesis of a three-step model of the cleavage process. The first step is the formation of the enzyme-substrate (ES) complex (Fig. 1, species 1), and it has been proposed that the enzyme may use active site interactions to orient the β-lactam carbonyl carbon near the Ser-70 nucleophile [19, 40]. The next step proposed along the reaction coordinate is the nucleophilic attack of Ser-70, which results in the opening of the β-lactam ring and the formation of the covalently bound active site acyl intermediate (species 3). For cephalosporins there is evidence that during the enzymatic reaction a leaving group (denoted R in Fig. 1) is split off [44, 45]. In the third step, the open-ring β-lactam ligand is hydrolyzed and released by the enzyme (Fig. 1, species 4). Various rates have been reported for this step of the catalytic reaction across different classes of β-lactams, followed by product release [40]. Static structures of some of the critical intermediates have been determined and reported [19], including an initial enzyme substrate complex trapped by removal of catalytically important amino acid residues [39]. Obtaining time-resolved data on BlaC chemistry holds the potential to directly visualize substrate chemical intermediates and the accompanying active site interactions, with wide-ranging implications for all classes of β-lactams. Ultimately, knowledge of the molecular processes by which BlaC is able to bind and catalyze the breakdown of β-lactams will directly impact rational drug design against deadly human diseases.

**Fig. 2:**
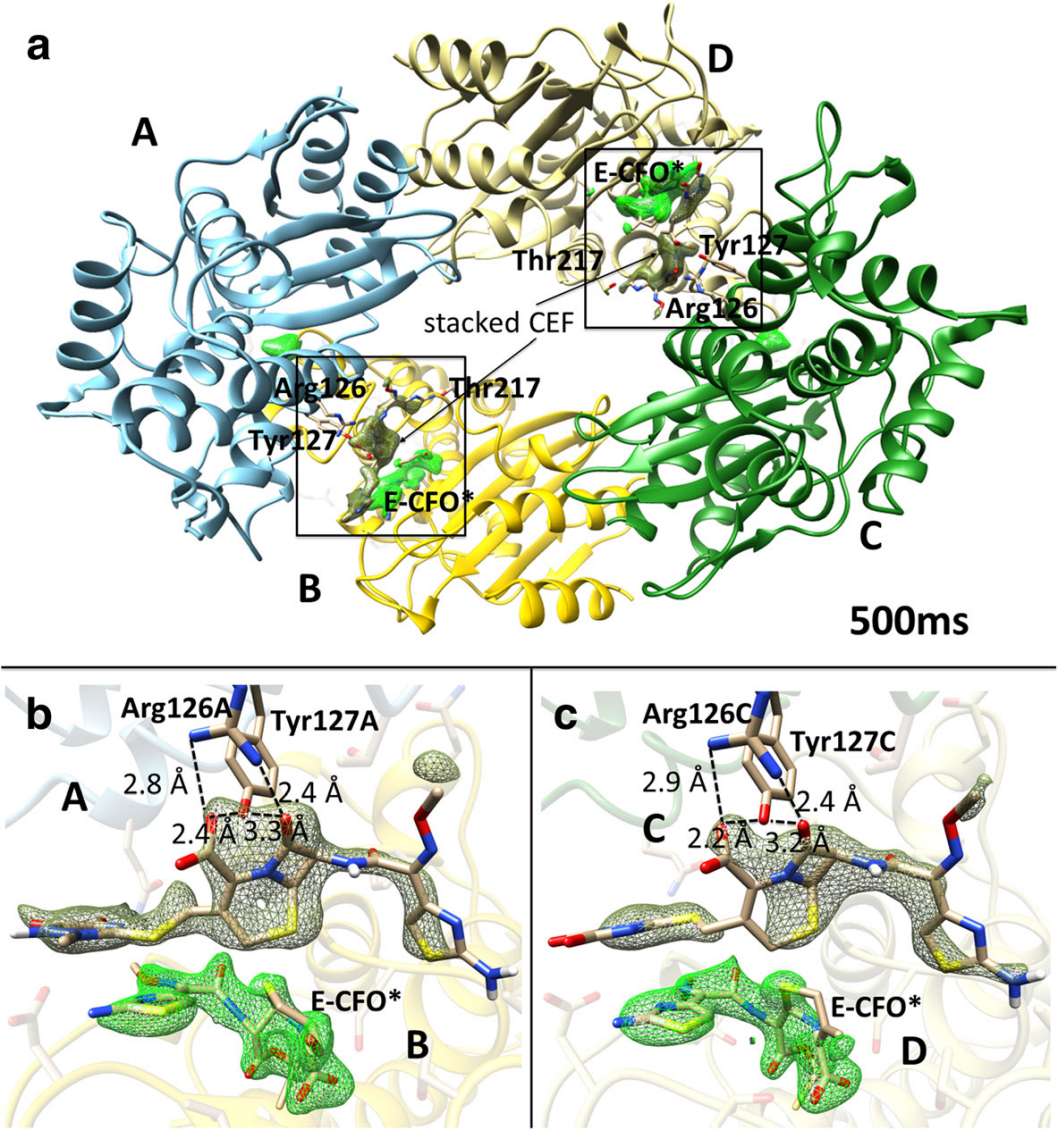
Overview of BlaC as determined using 10 × 10 × 3 μm^3^ sized crystals in the shard form at 500 ms after mixing with 300 mM CEF at room temperature. The mFo-DFc SA-omit electron density is shown for the covalently bound intermediate E-CFO* in *green* (contour level 2.5 σ). Electron density of an additional, stacked ceftriaxone molecule near the active site is shown in *dark green* (contour level 2 σ). **a** The BlaC subunits A–D displayed in *blue*, *yellow*, *green* and *light yellow*, respectively. Amino acid residues that interact with the stacked CEF are labeled. Panels **b** and **c** show enlarged views of the active sites of subunits B and D, respectively. Arg-126 and Tyr-127 with which the respective stacked CEF molecules interact are shown. Some important distances are also displayed (stacked molecules are also observed at the other time delays in the shard crystal form but not in the needles)

Our previous results at 2 s after mixing showed that CEF can diffuse into the crystals and binds to the active site of the crystalline β-lactamase [30]. These first studies showed that the catalytic reaction is heterogeneous, as the reactivity is specific to individual copies of the four β-lactamase chains in the asymmetric unit of the crystal. Only subunits B and D bind and process CEF, while subunits A and C do not directly contribute to catalysis, at least on the time scale of our experiments (Fig. 2a). This first proof-of-concept study was limited to a single time point about 2 s after reaction initiation [30]. Multiple time points covering the reaction are required for any kinetic analysis.

## Results

Here we present a time series from 30 ms to 2 s after mixing with substrate in two different crystal forms, called shards and needles; this allows us to discover the conformational changes and to characterize the kinetics of this important class of enzymes directly from the X-ray data. We base our interpretation on bias-free omit maps obtained by simulated annealing. Figure 2 and Additional file 1: Figures S2–S6 show details of these maps near the active site. As a complement, more conventional 2mFo-Fc maps are shown in Additional file 1: Figures S7–S9.

The critical questions in MISC concern whether the enzyme in the crystals is still catalytically active and whether the reaction is limited by constraints of crystal packing or the solvent/precipitant used for crystallization. We have therefore crystallized BlaC in two different crystal forms. With phosphate as the precipitant, the BlaC crystallizes in a shard-shaped crystal form with four copies in the asymmetric unit (Fig. 2a) as previously reported [30]. With polyethylene glycol (PEG) 1000 as the precipitant, needle-shaped crystals are obtained with one molecule in the asymmetric unit. The packing of BlaC in both crystal forms is shown in Additional file 1: Figure S11.

**Fig. 3:**
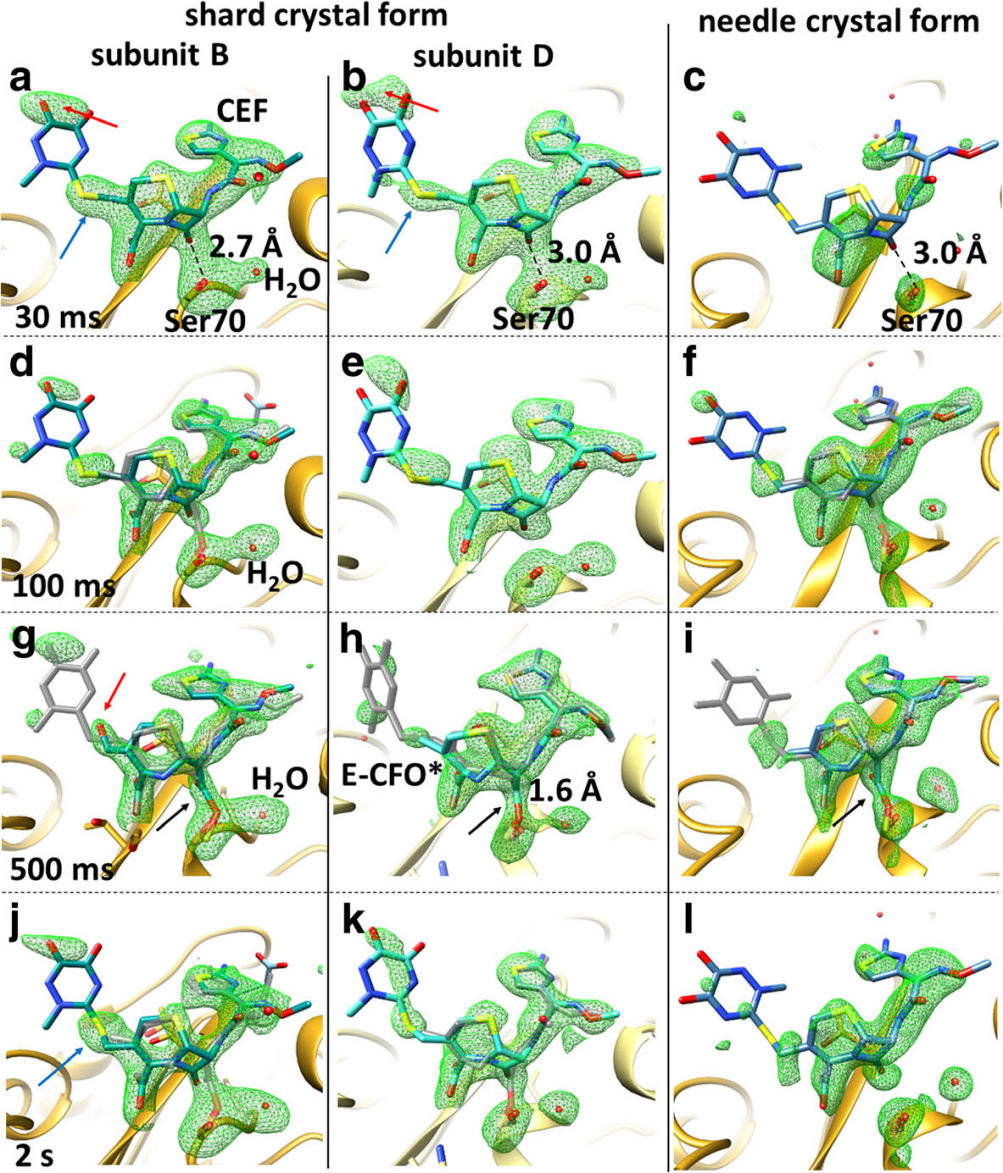
Ceftriaxone density in the active site in 10 × 10 × 3 μm^3^ shard and 5 × 2 × 2 μm^3^ needle crystal forms at various times after mixing with 200–300 mmol/L CEF. The main species is displayed in *blue*, the minor species in *gray*. *First two columns*: shard crystal form, mFo-DFc SA-omit density (*green*) contoured at 2.5 σ. *Third column*: needle crystal form. SA omit maps were calculated using extrapolated structure factors. Time delays are arranged from top (30 ms) to bottom (2 s). *Black arrows* show the electron density of the covalently bound acyl adduct (see also Additional file 1: Figure S2 for details). **a**, **b**, **c** The ES complex at 30 ms. The full-length CEF model (*blue*) is displayed. The ES complex can be observed in needles or shards (both subunits). *Blue arrows*: features of the leaving group sulfur, *red arrows*: dioxo-triaxine ring feature. **d**, **e**, **f** Early phases of the formation of a covalently bound CEF adduct at 100 ms. The full-length CEF model (*blue*) is displayed together with the minor E-CFO* species (*gray*), where the β-lactam ring is open and attached to Ser-70 in subunit-B (shard crystal form, panel **d**) and the needle crystal form (panel **f**). In the shard crystal form subunit D (panel **e**) the acyl adduct is not yet observed. **g**, **h**, **i** Covalently bound adduct (E-CFO* in blue) formation at 500 ms with a small contamination of full-length CEF (*gray*). The *red arrow* points to electron density that may favor the interpretation by an OH group. **j**, **k**, **l** Mixture of the non-covalently bound, full-length CEF (*blue arrow* shows the leaving group sulfur feature) and covalently bound E-CFO* in the shard crystal form (both subunits) at 2 s. The electron density in the needle crystal form favors only the full-length CEF species

In our MISC experiment, the small microcrystals were rapidly mixed with CEF “on the fly” using optimized mixing devices (Additional file 1: Figure S1), and structures of the reaction of BlaC with CEF were determined by scattering from femtosecond X-ray pulses at five time points (unmixed, and 30 ms, 100 ms, 500 ms, and 2 s after mixing, respectively) during the reaction in both crystal forms. Results are shown in Figs. 1 and 2. CEF binds to the active site of BlaC as shown in Fig. 2a. In Fig. 3 more details are shown for the substrate binding in the shard and needle crystal forms (see also Additional file 1: Figure S2 for details from another viewing direction, and Additional file 1: Figures S3–S10 for stereo representations of various viewing directions and time points). Strong electron density at 30 ms shows that substrate diffusion into the crystals was successful. At this time delay the formation of the non-covalently bound ES complex is observed (Fig. 3a, b, c). The ES complex can be identified by strong electron density of the leaving group sulfur (blue arrows in Fig. 3a, b), and somewhat stronger dioxo-triazine ring features (red arrows in Fig. 3a, b). Since the resolution of our X-ray data at the 30-ms time delay is limited to 2.75 Å, the distinction between a non-covalently bound species and a covalently bound species (see below) is difficult. However, the non-covalently bound species dominates occupancy refinements (see Table 2a, and remarks therein). At 100 ms the ES complex still prevails and is the major component observed (~ 70%, see also Table 2). A minor fraction (~ 30%) has an open β-lactam ring (Fig. 3d, e, f). The open, covalently bound species E-CFO* can be identified more clearly at 500 ms, where it dominates the electron density (Fig. 3g, h, i). Only on a time scale longer than 100 ms does the nucleophilic attack of Ser-70 open the β-lactam ring. At 500 ms this results in high occupancy of an intermediate which is covalently bound to the enzyme called E-CFO* as shown in Fig. 3g, h, i. At the same time the leaving group R (Fig. 1) is split off, as witnessed by the vanishing density of the leaving group sulfur and some of the weak ring density features (compare Fig. 3d and g, or Fig. 3e and h; see also feature β in Additional file 1: Figure S10c). The covalently bound ligand is much shorter than CEF. The red arrow in Fig. 3g indicates that the double bond ∆ (Fig. 1) may have reacted to an alcohol in subunit B, which does not occur in subunit D or in the needle form of the crystals. Additional file 1: Figure S10 shows the density in the unmixed shard crystal form (Additional file 1: Figure S10a) and a difference map between the 500 ms and the 100 ms time points (Additional file 1: Figure S10c), which displays changes in the region of the covalent attachment of the intermediate between 100 ms and 500 ms. At 2 s, the binding sites are occupied mainly by the full-length CEF with a minor contribution from E-CFO* (Table 2a, b).

**Fig. 4:**
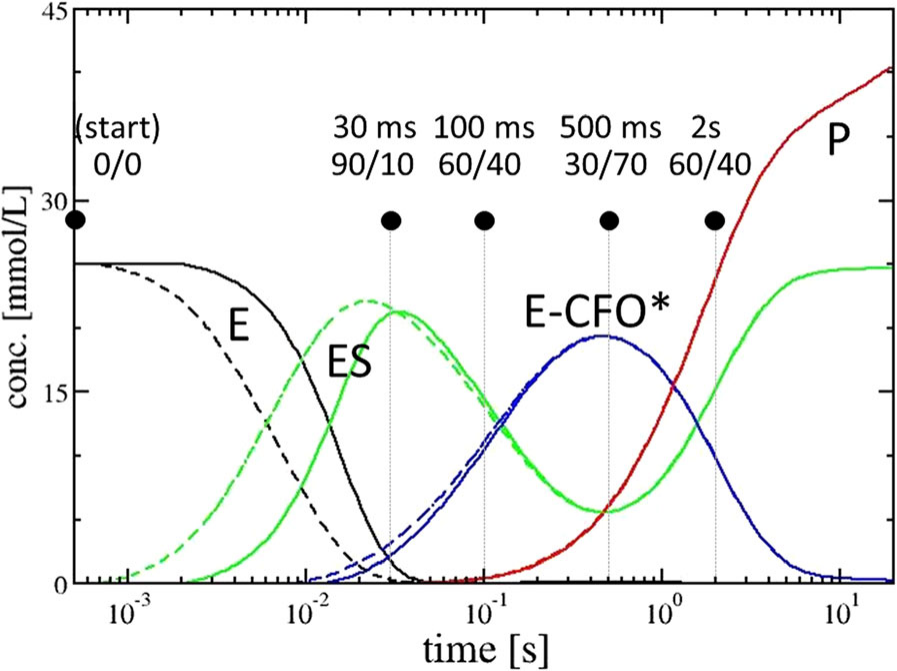
Concentration profile of the catalytic BlaC reaction with CEF as simulated with realistic parameters and a kinetic mechanism as discussed. The *solid lines* are calculated with τ_D_ = 15 ms, the *dashed lines* with τ_D_ = 1.5 ms. *Black lines*: free enzyme (*E*). *Green lines*: enzyme substrate complex (*ES*). *Blue lines*: enzyme acyl intermediate complex (E-CFO*). *Red lines*: product *P* (CFO, inactive CEF without leaving group, lactam ring open), released from the enzyme. *Black dots*: time delays are shown together with the approximate expected ratio of CEF to E-CFO*

In the multi-copy shard crystal form, subunits A and C do not directly participate in catalysis, at least not in the first 2 s. In the monomeric needle crystal form, it appears that the reaction proceeds similarly to that observed in subunit D in the shards. However, substrate occupancy is lower than that in the shards, with substoichiometric occupancy ranging from 20% to 40%. The reason for this might be that the enzyme is more tightly packed in the needle crystal form (Additional file 1: Figure S11). To reach full occupancy in the needles, at least 30 mmol/L of CEF (one CEF molecule per asymmetric unit) is initially required, which needs to be delivered by diffusion from the solution to the side of the crystal. While the outside CEF concentration is on the order of 200 mmol/L in both experiments (Table 3c), the ratio of CEF to enzyme varies in the shard and needle crystals. Additional file 1: Figure S11 shows how the solvent volume that contains CEF surrounding the BlaC molecules in the crystals varies. The solvent volume as estimated by the CCP4 program “truncate” [46] is on the order of 59% for the shard-shaped crystal, and it is substantially lower (28%) in the needles. Additional file 1: Figure S11 also shows that there are substantial differences in the solvent channel sizes in the two crystal forms. When measured by the program Coot [47], cavities with diameters as wide as 90 Å can be identified in the shards, and only 20-Å voids exist in the needles. Both may significantly impact diffusion of the CEF substrate (which is about 20 Å long and 10 Å wide) into the crystals. However, compared to other antibiotics such as ampicillin [40], CEF binds relatively slowly to BlaC, so it may first build up by diffusion (see also the discussion below and estimates in the Methods section). Strong electron density in our maps shows that diffusion and formation of the ES complex is near completion on a 30-ms time scale. Diffusion times and time scales of the ES formation (30 ms) are irrelevant compared to those for the E-CFO* intermediate formation (~ 500 ms). As a consequence, the ability to observe the E-CFO* intermediate does not critically depend on CEF diffusion times into the crystals (compare the solid and dashed lines in Fig. 4). Accordingly, the reaction dynamics of the catalytic reaction in the needle and the shard crystal forms appear to be similar despite the different crystal morphologies and packing (see further explanation in the Methods section). Subtle differences between the results from the two crystal forms, and between the subunits in different crystal environments, confirm previous preliminary results [30] and previous predictions from biochemical results for other cephalosporin species [44].

An additional CEF molecule (CEF^stack^) can be identified near the catalytic clefts of subunits B and D, each in the shard crystal form (Fig. 2a, b, c, and Additional file 1: Figure S9). This molecule stacks to the CEF species that occupy the active sites on all time scales. CEF^stack^ is non-covalently interacting with Arg-126 and Tyr-127 of subunit A or C, which are adjacent to the active catalytic clefts of subunit B or D, respectively. For more details see Additional file 1: Figure S9 for a stereo view. This way CEF^stack^ is quite close, pre-oriented, and can rapidly access the active site after the initial CFO has been hydrolyzed and has left the enzyme. Stacking of multiple cephalosporin (cefamandole) molecules has also been observed in orthorhombic crystals of the BlaC K73A mutant [39]. In these crystals the stacked molecules interact with Asp-192 and Arg-194 of a symmetrically equivalent BlaC molecule; this is different from the interactions seen here. As with most proteins, BlaC can crystallize in crystal forms with different numbers of copies in the asymmetric unit [39, 48, 49]. Since stacking is not observed in crystals that pack in the same way as our needle crystal form [19], it might be argued that it represents a non-physiological, non-specifically bound substrate that occurs only in the environment in the shard crystals. However, the binding of the additional CEF molecule could be a mechanism to steer the substrate towards, and orient it with respect to, the active site under certain conditions. It appears, however, that at the very high concentrations of CEF applied here (around 200 mmol/L), stacking is not required for effective catalysis, as the kinetics in the monomeric needles, where stacking does not occur, is similar to that in the shard crystal form. When only low CEF concentrations are present, stacking might well be essential to recruit antibiotic substrate molecules to promote effective BlaC function.

## Discussion

**Scheme 1:**
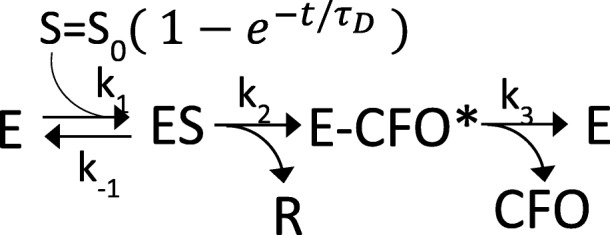
ᅟ

One of the major questions addressed here is whether the structural data obtained by MISC can be interpreted in accordance with previous investigations on BlaC catalysis. Ideally, a compatible chemical kinetic mechanism can be developed and expressed in the terminology of enzyme kinetics [50, 51]. Accordingly, we set up a kinetic mechanism (see Methods, Scheme 1) which allows for diffusion of substrate and which features a sufficiently large number of intermediate states to explain our observations. Initially, we simulated the catalytic cycle employing literature values of the Michaelis constant *K*_m_ (~ 500 μmol/L) and *k*_cat_ (0.8 s^− 1^) [40] (Table 3). Our simulations describe the change from the transient state kinetics regime at 30 ms to 2 s, covering a large range of substrate and product concentrations. We then vary the rate coefficients in the mechanism to explain our MISC experiment. Since only five time delays are available, the parameters in the mechanism cannot be independently determined, but we do show that our interpretation is consistent with known kinetic parameters in solution. After initial formation of the ES complex represented by a non-covalently bound full-length CEF, the intermediate E-CFO* has its peak concentration at 500 ms. It has been previously suggested [19] that the hydrolytic cleavage of an acyl adduct from Ser-70 (hydrolysis of species 3 in Fig. 1) should be the rate-limiting step in BlaC catalysis. Then the E-CFO* species should be the dominant species in the steady state. However, this is not the case, as the ES complex with the non-covalently bound, full-length CEF is prevalent (> 70%) in our MISC data at 2 s (Table 2). The simulation can explain this, if the nucleophilic attack of Ser-70 on species 2 in Fig. 1 is inhibited or slowed down. High product concentrations of > 10 mmol/L are already reached after one catalytic cycle due to the very high enzyme and substrate concentrations (Table 3). In initial velocity solution studies, the enzyme concentration is kept in the low micromolar range. Even under a saturating substrate (*v*_max_) condition, only micromolar concentrations of product can be produced per turnover. With a turnover rate of 0.8 s^−1^ of BlaC, it may take hours to reach millimolar concentrations of product. In BlaC crystals and with stoichiometric CEF concentrations (of 16 mmol/L for the shard crystal form, and 30 mmol/L for the needles), however, these concentrations are already reached after one turnover. Consequently, product inhibition is not only plausible but likely, as previous studies have shown that lactamases do show product inhibition by similar lactams with inhibitory constants in the millimolar range [52]. In this scenario, after an initial burst during the first second, the nucleophilic attack on the lactam ring by Ser-70 represented by rate coefficient *k*_2_ likely becomes the rate-limiting process (the E-CFO* formation slows down), and the ES complex accumulates later, as observed in our X-ray data.

Our results depend decisively on the ability of CEF to penetrate the crystals. Although pore sizes for shard and needle crystal forms largely differ (see above), CEF substrate swiftly arrives at BlaC molecules in the crystals. This is primarily due to the large substrate concentrations that facilitate diffusion (see Methods and Table 3). The osmotic pressure *π* of the outside CEF into the crystals can be estimated as *π* = *MRT*, where *M* is the molarity of the outside CEF concentration, *R* is the gas constant, and *T* is the temperature in kelvins. This pressure can be as high as 0.5 MPa (5 bar) with 300 mmol/L CEF, which promotes rapid and uniform diffusion, even in crystals with tight cavities such as those in our needles (Table 3c). Diffusion may also be further enabled, and facilitated, by protein dynamics [53] at ambient temperatures. Although the reaction kinetics in crystals might be different compared to that in solution [54], structures of intermediate states that are occupied along the catalytic pathway are highly relevant as long as the enzyme is active in the crystal. With more conventional X-ray sources, radiation damage might impede the collection of even a single diffraction pattern [55] from these microcrystals. The ultrashort, brilliant hard X-ray pulses available at XFELs circumvent these difficulties. With high X-ray pulse-repetition rates expected at LCLS-II [56] and the European XFEL [57], a large number of finely spaced time delays may be collected rapidly to allow for a comprehensive description of the reaction in terms of structure and kinetics. Then the extraction of a more accurate kinetic mechanism and the analytical separation of mixtures into pure constituents become possible [4, 58].

## Conclusions

As we demonstrate here, the structural characterization of enzyme-catalyzed reactions on the millisecond time scale is possible by making use of very small crystals. MISC can be employed to investigate a large number of non-cyclic (single-pass) reactions in proteins and enzymes, some of which are of immense biological significance and might be important targets for structure-based drug design. MISC may become a major tool to address fundamental questions on biomolecular reactions at existing and new pulsed X-ray sources.

## Methods

### General overview

Using a continuous-flow mixing apparatus (Additional file 1: Figure S1), we injected active microcrystals of BlaC simultaneously with the β-lactam substrate ceftriaxone (CEF) into a liquid jet for delivery to the beam as a stream of randomly oriented hydrated nanocrystals *undergoing catalysis*. The catalytic reaction is initiated by solution mixing at the junction of two capillaries [38] and the nanocrystals intersected by the X-ray pulse at specific time points during the reaction. The use of nanocrystals is essential for observation at short times and for effective and uniform reaction initiation [28]. The 120-Hz repetition rate of LCLS allowed for the rapid collection of diffraction snapshots at a number of delay times (time points) after reaction initiation. Accurate reflection intensities were extracted from the snapshots at each time point by indexing and Monte Carlo-type integration [59, 60]. The data were phased using the structural model for BlaC reported by Kupitz et al. [30]. This model is based on the BlaC Protein Data Bank (PDB) entry 2GDN [48]. The sequence convention reported in PDB entry 2GDN has also been used by others [19], and we use it here throughout for homogeneity. Accordingly, we obtained, as a function of time, information on distinct chemical intermediates of β-lactam substrates within the active site of BlaC. The BlaC enzyme requires limited conformational changes to execute catalysis, allowing us to observe the full enzymatic reaction within a crystal.

### Crystal forms

Cloning, overexpression, and purification of *M. tuberculosis* BlaC was performed as described previously [30]. BlaC was crystallized in the shard crystal form as described earlier [30]. The slurry was stirred overnight at 30 °C to avoid the growth of larger crystals that otherwise need to be crushed to be suitable for MISC experiments. Crystals grown this way were of dimensions 10 × 10 × 3 μm^3^. An additional crystal form was obtained from a different crystallization condition using the free interface diffusion (FID) method [61]. In a 1.5-mL Eppendorf tube, 250 μL of a precipitant solution (35% PEG 1000, sodium acetate pH 5.0), was slowly added dropwise through 250 μL of a protein solution at 10 mg/mL. Needle-shaped crystals of dimensions 5 × 2 × 2 μm^3^ grew at room temperature in about 48 h. The microcrystalline sample was highly monodisperse as demonstrated by dynamic light scattering (Additional file 1: Figure S12). The suspension showed an intense second order, non-linear imaging of chiral crystals (SONICC) signal demonstrating the crystallinity of the sample. X-ray powder diffraction was used as a quality test to verify the existence of diffracting crystals. A very high-density pellet of microcrystals was transferred to a transparent plastic capillary (MiTiGen, Ithaca, NY, USA). A small amount of precipitant solution was kept to prevent the crystals from drying out. The capillary was mounted onto a regular goniometer base, and data were collected for 3 min on a Rigaku Micro Focus 007 high-flux X-ray generator. Intense powder rings were observed up to 7 Å. Weaker rings were also observed to extend up to approximately 4 Å.

### Injectors

The mixing injectors used in this experiment were based on the design by Calvey et al. [36] shown in Additional file 1: Figure S1. In these devices, a crystal suspension and a buffer (either 1 mol/L sodium phosphate or sodium acetate, pH 5) containing 200–300 mmol/L CEF are flowing in coaxial capillaries. The flows are combined and forced into a constriction, thinning the crystal flow to a narrow jet and allowing rapid CEF diffusion. By varying the length of the device, the sample and buffer flow rates, or by placing an expanded region after the constriction, we were able to probe time scales ranging from 30 ms to 2000 ms. Two high-performance liquid chromatography (HPLC) pumps (Shimadzu LC-20 AD) drove the flow. Crystals (shards: 10 × 10 × 3 μm^3^, needles: 5 × 2 × 2 μm^3^) were held in a custom reservoir built by Coherent X-ray Imaging (CXI) staff, while the buffer was held in a larger reservoir (KNAUER VariLoop), allowing water flow through the HPLC pump without diluting either sample or buffer. A pressure controller (Proportion-Air GP1) was used to regulate helium pressure in the device. For each condition, the solution is considered mixed when the CEF concentration exceeds 40 mM, which is sufficiently high to cause rapid binding. The reported mixing times are the time for the concentration around the average crystal to reach this threshold, with upper and lower bounds given for the first and third quartiles. In these calculations, the crystals are assumed to be much smaller than the focused jet, and fluctuations in flow rate are neglected. The mixing times for each time point are reported in Table 1. The delay time is defined as the time that the reaction is allowed to proceed after mixing. During this time, the crystals traverse the device before being probed by the X-ray beam. Uncertainty in the delay time results from errors in the sample and buffer flow rates (which come from the factory specifications for the Shimadzu LC-20 AD HPLC pumps that we used to drive the flows) and from small variations in the diameters and lengths of the capillaries used to make the mixing injectors. Mixing injectors were designed so that the delay time slightly exceeded the nominal time point to allow for additional time for the ceftriaxone to diffuse into the crystals. Table 1 lists the delay times and flow parameters for different time points.

**Table 1:** Mixing parameters for each time point. The buffer contained 200–300 mmol/L CEF in either 1.0 mol/L sodium phosphate (shard crystal form), pH 5, or in 100 mmol/L sodium acetate, pH 5 (needle crystal form). The superscript and subscript numbers in the second column indicate that deviations to shorter times are different from those to longer times

Nominal time point (ms)	Mixing time (ms)	Delay time (ms)	Sample flow (μL/min)	Buffer flow (μL/min)	Constriction diameter (μm)
30	${5}_{-3}^{+6}$	42 ± 2	4.0 ± 0.5	66.0 ± 0.6	75 ± 1
100	${10}_{-8}^{+13}$	114 ± 4	10.0 ± 0.5	70.0 ± 0.7	75 ± 1
500	${7}_{-5}^{+10}$	510 ± 20	8.0 ± 0.5	32.0 ± 0.5	50 ± 1
2000	${15}_{-11}^{+20}$	2300 ± 50	10.0 ± 0.5	45.0 ± 0.5	75 ± 1

### Data collection, data analysis, and structure determination

Serial femtosecond crystallography (SFX) experiments were performed at the CXI instrument [62]. Microcrystals (10 × 10 × 3 μm^3^ shard-shaped crystals or 5 × 2 × 2 μm^3^ needles) were mixed with the antibiotic ceftriaxone (200–300 mmol/L) before injection into a vacuum using a mixing jet injector (described above) that allowed millisecond time resolution. Diffraction patterns were recorded on a Cornell-Stanford Linear Accelerator Center (SLAC) pixel array detector (CSPAD) [63] operating at 120 Hz to match the X-ray pulse frequency. Data for shards and needles were analyzed in an identical fashion. Cheetah [64] was used to filter out diffraction patterns containing Bragg reflections. These patterns were indexed and integrated using the CrystFEL (version 0.6.2) program suite [60, 65]. Partial intensities were scaled and merged using linear and Debye-Waller factor scale factors. Data statistics are listed in Table 2. The BlaC structures were solved for the needles and shards using molecular replacement by Phaser [66]. For the shards, the structure determined by Kupitz et al. [30] with four subunits (A–D) in the asymmetric unit was used as the initial model. For the monomeric structure in the needles, subunit D from this structure was extracted and used as a search model. Reference structures S_ref,n_ and S_ref,s_ were determined for the needles and shards, respectively, using the respective “unmixed” data for both crystal forms. To determine structural changes after mixing, difference maps were determined. For the shards, unit cell changes on the order of 2 Å and larger were observed after mixing. This prevents the calculation of isomorphous difference maps. With the needles, however, unit cell changes were not observed (Table 2), and isomorphous difference maps can be calculated. Accordingly, two different strategies were followed to analyze the two types of data.

1. *Structures for the shard crystal form*. Since isomorphous difference maps could not be calculated, structural interpretation has been based on omit difference maps. The reference model was refined using simulated annealing (SA) in PHENIX against the observed $\left|{F}_t^{obs}\right|$. For this refinement, water and phosphate molecules residing in the active sites of all subunits were removed. In addition Ser-70 was replaced by a glycine (Gly-70) in subunits B and D. The structure was heated to 5000 K (default) and slowly cooled to 300 K. As a result, a model of the *apo*-protein without any ligands in the active site was obtained. After the refinement, mFo-DFc omit difference maps ${\Delta  \rho}_t^{omit}$ were calculated for each time point *t*, where the Fo correspond to the $\left|{F}_t^{obs}\right|$ and the Fc are determined from the refined (partial) model, m is the figure of merit, and D is a coordinate error-dependent weighting term [67, 68]. The resulting omit map is essentially free of phase bias towards the ligand-free “unmixed” structure.

**Table 2:** Data collection and refinement statistics

	Reference	30 ms	100 ms	500 ms	2 s
(a) Shard-shaped crystals					
Hits	98,895	35,065	88,413	158,620	39,140
Indexed images	73,170	24,397	79,328	134,583	32,201
Resolution (Å)	2.45	2.75	2.15	2.20	2.30
Space group	P2_1_	P2_1_	P2_1_	P2_1_	P2_1_
Unit cell (Å,^o^) (a, b, c, and β)	79.0 97.2 110.6 108.7	78.7 96.8 112.6 109.7	79.2 96.5 113.7 109.9	78.8 96.3 113.5 110.0	78.2 95.6 112.3109.9
Volume (Å^3^)	804,442	807,597	817,098	809,346	789,415
BlaC/unit cell	8	8	8	8	8
Completeness	100(100)	100(100)	100(100)	100(100)	100(100)
Multiplicity	1221 (103.3)	526 (142.0)	895 (58.8)	1363 (81.3)	330 (59.0)
SNR	8.9(2.4)	6.4(0.9)	7.1(1.0)	8.3(0.9)	5.4(1.1)
R_split_ (%)	9.8(209.4)	14.2(121.1)	11.18(111.0)	9.7(126.3)	11.9(104.1)
CC-half (%)	99.4(41.1)	98.6(34.5)	99.4(37.5)	99.6(31.0)	96.8(35.4)
Refinement					
R_cryst_/R_free_ (%)	19.2/24.4	19.3/25.0	20.9/23.9	21.9/25.0	23.5/26.6
^*B^CEF/E-CFO*^a^	0/0	91 (23^b^)	57/32	40/36^c^	58/25
^*D^CEF/E-CFO*^a^	0/0	89 (24^b^)	54/40	38/44	51/31
Stacking	no	yes	yes	yes	yes
H_2_O	315	143	499	431	399
Average B value (Å^2^)	48.2	51.7	42.3	37.3	36.2
Protein amino acid residues in asym. unit	265 × 4	265 × 4	265 × 4	265 × 4	265 × 4
Ligands	0	2 + 2 (stacking)	2 + 2 (stacking)	2 + 2 (stacking)	2 + 2 (stacking)
RMSD bond lengths (Å)	0.008	0.010	0.008	0.008	0.008
RMSD bond angles (degrees)	1.10	1.72	1.66	1.67	1.74
PO_4_	4	2	2	2	2
(b) Needle-shaped crystals					
Hits	171,314	64,507	115,223	141,935	36,606
Indexed images	111,466	34,590	87,580	87,058	23,278
Resolution (Å)	1.8	1.9	1.8	1.9	2.05
Space group	P2_1_	P2_1_	P2_1_	P2_1_	P2_1_
Unit cell (Å,^o^) (a, b, c, and β)	39.6 41.6 69.3 104.8	39.5 41.6 69.3 104.8	39.6 41.6 69.3 104.9	39.6 41.7 69.5 104.9	39.6 41.7 69.5 104.9
Volume (Å^3^)	110,375	110,096	110,323	110,908	110,908
Completeness (%)	100(100)	100(100)	100(100)	100(100)	100(100)
Multiplicity	985 (54.5)	330 (26.8)	831 (89.0)	806 (36.5)	238 (27.3)
Signal-to-noise ratio	9.6(1.2)	5.8(0.8)	9.6(1.6)	8.6(0.9)	5.1(1.1)
R_split_ (%)	6.6(97.0)	12.2(136.3)	6.6(72.5)	8.8(129.1)	14.0(105.9)
CC* (%)	99.9(75.0)	99.9(76.1)	99.9(84.3)	99.9(68.1)	99.8(74.8)
CC-half (%)	99.7(39.1)	99.4(40.4)	99.7(55.1)	99.7(30.2)	99.13(38.8)
Refinement					
R_cryst_/R_free_ (%)	21.5/24.5	20.7/26.2	23.0/26.7	21.7/26.4	20.0/25.0
N^d^	na	9	9	6	5
CEF/E-CFO*^a^	0/0	59/0	51/35	43/53	71/0
Stacking	no	no	no	no	no
H_2_O	167	203	154	104	175
Average B value (Å^2^)	34.7	16.9	10.5	15.7	18.3
Protein amino acid residues in asym. unit	265	265	265	265	265
Ligands	0	1	1	1	1
RMSD bond lengths (Å)	0.008	0.007	0.007	0.003	0.008
RMSD bond angles (degrees)	1.06	1.57	1.74	1.49	1.57

^*B^for subunit B

^*D^for subunit D

^a^occupancy of full-length, intact CEF to covalently bound, open E-CFO*, which has lost R. Numbers are rough estimates and should represent only trends (the error is on the order of 25%, see note b)

^b^omit maps show only CEF in the active site. If E-CFO*‘s occupancy is refined at the same time, values around 24% are obtained. We consider this the error of our occupancy refinement

^c^Addition of OH instead of the double bond ∆

^d^If N does not extrapolate to 100% occupancy, a fraction of reference structure is present. This is ignored in the refinement

*na* not applicable

*RMSD* root mean square deviation

Strong electron density appeared in subunits B and D that was reminiscent of CEF molecules. In subunits A and C, the electron density of only the phosphate and the water molecules re-appeared, a result that was also previously reported [30]. Hence, the structures of the catalytic clefts in these subunits A and C were restored back to the reference. The ${\Delta  \rho}_t^{omit}$ in the catalytic clefts of subunits B and D was exceptionally strong at all time delays (Fig. 3, Additional file 1: Figures S2–S4, S6). Appropriate CEF species (Fig. 1) were placed in the positive ${\Delta  \rho}_t^{omit}$ and initially real-space refined in Coot using adequate crystallographic information files (CIFs), which define the geometry and provide restraints. CIFs were generated for the full-length ceftriaxone (CEF) as well as an open form with the leaving group split off (E-CFO*) as previously described [30]; compare also Fig. 1. One oxygen of the open lactam carboxyl in E-CFO* was removed, and the carboxyl carbon was connected to the Ser70-O_g_ with a weak distance restraint of 1.6 Å. At all time points, either CEF, E-CFO* (bound to Ser-70), or a mixture of both was observed. Their structures were first refined in real space in Coot.

Mixtures of full-length, non-covalently bound CEF configurations and Ser-70 bound, open forms (CFO) were refined together in PHENIX. Note that E-CFO* was replaced at 500 ms in subunit B with a species displaying an alcohol (Figs. 1d, 3g and Additional file 1: Figure S6b) instead of the double bond ∆, the structure of which was refined as described. Further refinement including occupancy refinement of the two species was performed with PHENIX [69] against the $\left|{F}_t^{obs}\right|$. Since a large volume of electron density is shared by CEF and the shorter E-CFO*, occupancy refinement is not reliable. Numbers obtained reflect the fact that the two molecules are present. Essentially complete ligand occupancy is reached at all time delays. Therefore, a potential presence of an unmixed BlaC species was not taken into account during the refinement. An additional CEF molecule, which can be identified near, but not bound to, the active site (CEF^stack^) has been added to the last phase of the refinement. The leaving group (the large dioxo-triazine ring) is π-π stacking with the small amino-thiazol ring of the CEF species in the active site, resulting in an antiparallel alignment. Distances between the rings are on the order of 3.5 Å. However, as mentioned later, the main interactions are with Tyr-127 (between Tyr-127O_η_ and O_I_ of the CEF^stack^ dihydrothiazine carboxyl) and Arg-126 (between Arg-126N_ε_ and O_I_ of CEF^stack^-O_I_) of the adjacent (non-reactive) dimer subunit (see Fig. 2b, c and Additional file 1: Figure S9). CEF^stack^ is pre-oriented this way very close to the active site. In order to access the active site, CEF^stack^ only has to flip in, which may be initiated when the CFO species leaves the active site. B-factors of the various CEF species in the shard crystal form are shown in Additional file 1: Table S1.

2. *Structures for the needle crystal form*. Difference structure factor amplitudes ${\Delta  F}_t^{iso}$ were calculated for each time point *t* by subtracting the observed reference structure factor amplitudes $\left|{F}_{ref}^{obs}\right|$collected with no CEF present from the time-dependent structure factor amplitudes $\left|{F}_t^{obs}\right|$. From the ${\Delta  F}_t^{iso}$ and the phases derived from S_ref,n_, isomorphous difference maps were calculated. In order to model the BlaC structure including a (potentially modified) CEF ligand, conventional electron density maps ${\rho}_t^{ext}$ were calculated where the ligand occupancy was extrapolated to 1.0. Extrapolated structure factors ${F}_t^{ext}$ were calculated by adding the ${\Delta  F}_t^{iso}$ *N* times (see Table 2) to the calculated structure factors derived from S_ref,n_. The extrapolated electron density ${\rho}_t^{ext}$ was derived from the ${F}_t^{ext}$. The structures of appropriate CEF derivatives (see above and Fig. 1) were inserted using Coot [47]. At all time points, either CEF, E-CFO* (bound to Ser-70), or a mixture of both was observed (Fig. 3c, f, i, l). Their structures were first refined in real space against the ${\rho}_t^{ext}$ in Coot. Further occupancy refinement was performed as described above (1).

### Enzyme kinetics

The Michaelis constant *K*_m_ is on the order of 500 μmol/L for BlaC with CEF, and *k*_cat_ in solution is 0.8 s^−1^ [40]. The *k*_off_ rate coefficient of dissociation of substrate from the active site as well as the diffusion coefficient of CEF in the crystals are unknown. Accordingly, we need to assume values that yield plausible results. When the *k*_off_ rate coefficient is assumed to be equal to the *k*_cat_ rate coefficient in solution, the *k*_on_ rate coefficient for the binding of CEF is ${k}_{on}=\frac{2{xk}_{cat}}{K_m}=\frac{2x0.8}{500\ x\ {10}^{-6}}\ L\ {mol}^{-1}{s}^{-1}=3200\ L\ {mol}^{-1}{s}^{-1}$. Assuming a diffusion coefficient of 2.3 × 10^−6^ cm^2^/s for CEF in water [70], characteristic diffusion times τ_D_ into the centers of the 10 × 10 × 3 μm^3^ shards and the 5 × 2 × 2 μm^3^ needles would be a few milliseconds (Table 3c) [28]. It should be mentioned here that diffusion times in crystals may be very different from, and much slower than, those in solution. At the characteristic diffusion time, $\left(1-\frac{1}{e}\right)$ or 63% of the outside CEF concentration is reached in the crystal center. Crystal suspensions are typically mixed 1:4 with large concentrations of CEF (between 200 mmol/L and 300 mmol/L). Mixing ratios can be up to 1:17 for the fastest mixing times (Table 1). As a result, the CEF solution is only slightly diluted after mixing. The CEF concentration is much higher than the concentration of BlaC molecules in the crystals (16 mmol/L in the shard crystal form, 30 mmol/L in the needles). The stoichiometric concentration of CEF is reached at a time *t*, much faster than τ_D_. This time *t* can be estimated as *t* = *τ*_*D*_ ∙ f where *f* = $-\ln \left(1-\frac{s}{out}\right)$, *s* is the concentration of BlaC in the crystals, and *out* is the outside CEF concentration (Table 3c). *f* is ~ 0.1 for the shards and ~ 0.2 for the needles (see Table 3c). The experiment becomes robust towards variations in diffusion times caused by crystal size, crystal morphology, and crystal packing. Pore sizes in the shards (up to 90 Å) are up to four times larger than those in the needles (see discussion above, and also Additional file 1: Figure S11), which could severely impede diffusion, especially of a molecule as large as CEF (554.6 g/mol). As shown in Table 3c, diffusion times may be slower by two orders of magnitude compared to those in solution, and still the substrate would diffuse sufficiently fast to reach stoichiometric concentrations.

**Table 3:** Results from the simulations and estimates of diffusion times. (a) Parameters used in the simulation: apparent diffusion time τ_D_ based on crystal size, initial enzyme concentration *E*_0_, outside substrate concentration *S*_0_, rate coefficients *k*_1 …_ *k*_3_, and product inhibition *I*_p_ (Scheme 1). (b) Occupancy of the various enzyme species as obtained by the simulation. They can be compared to occupancy values listed in Table 2. *E* free enzyme, *ES* non-covalently bound ceftriaxone in the active site, with leaving group present, E-CFO* enzyme intermediate with CFO bound covalently, *P* free product (CFO). (c) Diffusion times τ_D_, and times *t* to reach stoichiometric concentration in the shard and needle crystals. As an example, estimates for the 1:4 (crystal:CEF) mixing ratio are listed. Time variations between 200 mmol/L and 300 mmol/L CEF are negligible. Times are lower limits, since they are estimated from diffusion in water. Even if they are allowed to vary by orders of magnitude, sufficient occupancy would be achieved after 30 ms. They also imply that the time resolution may be given by the mixing times (Table 1) in some crystal forms, and not by the diffusion times, since the former are longer than the latter

(a)							
_τD_ (ms)	*E*_0_ (mmol/L)	*S*_0_ (mmol/L)	*k*_1_ (L mmol^−1^ s^−1^)	*k*_−1_ (s^−1^)	*k*_2_ (s^−1^)	*k*_3_ (s^−1^)	*I*_P_ (mmol/L)
15/1.5	25	200	3.2	0.01	7	0.8	6
(b)							
	0 ms	30 ms*	100 ms	500 ms	2 s		
E (%)	100	10/2	2	1	1		
ES (%)	0	84	58	19	59		
E-CFO* (%)	0	6/14	40	80	40		
P ([mmol/L)	0	0.05	0.5	5.8	23.4		
(c)							
		Shard-shaped crystals		Needle-shaped crystals			
BlaC concentration in crystal		16 mmol/L		30 mmol/L			
Average size([μm^3^)		10 × 10 × 3		5 × 2 × 2			
τ_D_		3.5 ms		0.8 ms			
Mixing ratio crystal:CEF		1:4		1:4			
CEF = 200 mmol/L							
CEF after mixing		160 mmol/L		160 mmol/L			
Time to reach stoichiometric conc.		0.36 ms		0.17 ms			
CEF = 300 mmol/L							
CEF after mixing		240 mmol/L		240 mmol/L			
Time to reach stoichiometric conc.		0.24 ms		0.10 ms			

*For 15-ms and 1.5-ms diffusion times, respectively

The time-dependent concentrations of species along the enzymatic pathway were simulated by numerically integrating the coupled differential equations of the simple kinetic mechanism shown in Scheme 1 using the above rate coefficients which reproduce the known *K*_m_. Note that formation of the covalent E-CFO* complex (acyl intermediate) is irreversible due to the cleavage of the leaving group R from CEF. Table 3a lists the parameters that enter the calculation.

The substrate concentration *S* in the crystal is determined by the outside substrate concentration *S*_0_ (50 mmol/L in the simulation) and the characteristic diffusion time τ_D_. S is fed to the free enzyme E and bound to the enzyme with *k*_1_. The total enzyme concentration was set to 25 mmol/L. Results are shown in Fig. 4 and Table 3b for τ_D_ = 15 ms (solid lines) and for τ_D_ = 1.5 ms (dashed lines). Sufficient accumulation (occupancy) of the enzyme substrate complex (ES, green) is achieved after 30 ms even with the longer diffusion time (Fig. 4), which agrees with our observations by MISC (Fig. 3a, b, c). Initially, *k*_3_ was assumed to account for the rate-limiting process and set to *k*_cat_ = 0.8 s^− 1^. As the low *k*_3_/*K*_m_ ratio found in the literature [40] (~ 1.5 × 10^3^ L mol^−1^ s^−1^) suggests, CEF binds slowly to the enzyme. In Fig. 4, we show results for 1.5-ms as well as 15-ms diffusion times. As the kinetics are very similar, the MISC experiment is robust against crystal size heterogeneities as well as against mixing time jitter (Table 1). The ES complex accumulates slightly faster with the fast diffusion time, but the kinetics are essentially the same for both simulated diffusion times. The acyl intermediate (E-CFO^*^, blue) forms essentially on the same time scale (500 ms) for both crystal forms (Fig. 3 g, h, i). In our MISC X-ray data we do not see clear evidence of a product complex (EP) where the CFO has been hydrolyzed (detached) from Ser-70 and is non-covalently bound to the enzyme. It appears as if this product state is depopulated faster than it is populated, and it therefore does not accumulate sufficiently to be detected. Importantly, the ES complex reappears in our MISC data at 2 s (Fig. 3j, k, l). That means the E-CFO* cannot be the rate-limiting species (with the hydrolysis of the covalent bond the rate-limiting step); otherwise, E-CFO* would be the dominant species in the steady state. However, if ES were initially rate-limiting (and the nucleophilic attack of Ser-70 the rate-limiting step), E-CFO* would not accumulate sufficiently to be detected so clearly at 500 ms. To solve this dilemma, we assume that rate coefficient *k*_2_ (the Ser-70 nucleophilic attack) decreases with product concentration. Unlike in solution, in the crystal enzyme and substrate concentrations are so high that already after one turnover more than 10 mmol/L of substrate is converted to product. Accordingly, on time scales > 1 s, product inhibition was assumed by lowering *k*_2_:${k}_2={k}_2^{\prime}\left(1-{e}^{-{P}_n/{I}_p}\right)$, where *P*_n_ is the concentration of the released product P divided by an characteristic inhibitory concentration *I*_p_ in mmol/L (Table 3a). BlaC inhibition by penicilloic acids was also reported previously [52]. This detail of the BlaC reaction awaits further investigations which are outside the scope of this paper. By no means do we suggest that this mechanism is unique. There are only four time points (plus the unmixed, free enzyme species). The rate coefficients in the mechanism may vary widely and still reproduce the observations. Within a large number of plausible mechanisms, our mechanism is the simplest that explains our experimental observations at limited time points. If a more complex mechanism is to be justified, the collection of additional, more finely spaced time points is necessary.

## Additional file

Additional file 1: **Figure S1.** Schematics of the short-time-point mixing injector. **Figure S2.** Selected views of the CEF binding site in the BlaC shard crystals including simulated annealing omit maps. **Figure S3.** Structural details, and simulated annealing omit maps, shard crystal form, subunit B (stereo representation, from 30 ms to 2 s). **Figure S4.** Structural details and simulated annealing omit maps, shard crystal form, subunit D (stereo representation, from 30 ms to 2 s). **Figure S5.** Structural details, and simulated annealing omit maps, needle crystal form (stereo representation, from 30 ms to 2 s). **Figure S6.** Backside view of the catalytic cleft of BlaC in the shard crystal form, structural details and simulated annealing omit maps (stereo representation, selected time points). **Figure S7.** 2mFo-DFc electron density in the catalytic clefts of BlaC in the shard crystal form (stereo representation, from 30 ms to 2 s). **Figure S8.** 2mFo-DFc electron density and structural details in the catalytic clefts of BlaC in the needle crystal form (stereo representation from 30 ms to 2 s). **Figure S9.** Details in the catalytic cleft of subunit B in the shard crystal form at 500 ms including the stacked CEF, 2FoFc maps, and simulated annealing omit maps (stereo representation). **Figure S10.** The catalytic cleft of BlaC, further details, including a difference map between the 500 ms and 100 ms time points. **Figure S11.** Crystal packing in shards and needles. **Figure S12.** Dynamic light scattering results. **Table S1.** B-factors for CEF species observed in the shard crystals at different time delays. (PDF 1646 kb)

## Abbreviations

BlaC: *Mycobacterium tuberculosis* β-lactamase
CEF: Ceftriaxone
CFO: Ceftriaxone with lactam ring open and the leaving group split off
CSPAD: Cornell SLAC pixel area detector
CXI: Coherent X-ray imaging
E-CFO*: CFO species covalently bound to the enzyme
ES: Enzyme-substrate (complex)
FID: Free interface diffusion
*K*_m_: Michaelis constant
LCLS: Linac Coherent Light Source
MISC: Mix-and-inject serial crystallography
SLAC: Stanford Linear Accelerator Center
SFX: Serial femtosecond crystallography
XFEL: X-ray free-electron laser
