# Supplementary material for: Enzyme intermediates captured “on the fly” by mix-and-inject serial crystallography
Source: BMC Biol. 2018 May 31;16:59. doi: 10.1186/s12915-018-0524-5 (PMC5977757; doi:10.1186/s12915-018-0524-5)
Supplement: Supplementary file 1 — Figure S1. Schematics of the short-time-point mixing injector. Figure S2. Selected views of the CEF binding site in the BlaC shard crystals including simulated annealing omit maps. Figure S3. Structural details, and simulated annealing omit maps, shard crystal form, subunit B (stereo representation, from 30 ms to 2 s). Figure S4. Structural details and simulated annealing omit maps, shard crystal form, subunit D (stereo representation, from 30 ms to 2 s). Figure S5. Structural details, and simulated annealing omit maps, needle crystal form (stereo representation, from 30 ms to 2 s). Figure S6. Backside view of the catalytic cleft of BlaC in the shard crystal form, structural details and simulated annealing omit maps (stereo representation, selected time points). Figure S7. 2mFo-DFc electron density in the catalytic clefts of BlaC in the shard crystal form (stereo representation, from 30 ms to 2 s). Figure S8. 2mFo-DFc electron density and structural details in the catalytic clefts of BlaC in the needle crystal form (stereo representation from 30 ms to 2 s). Figure S9. Details in the catalytic cleft of subunit B in the shard crystal form at 500 ms including the stacked CEF, 2FoFc maps, and simulated annealing omit maps (stereo representation). Figure S10. The catalytic cleft of BlaC, further details, including a difference map between the 500 ms and 100 ms time points. Figure S11. Crystal packing in shards and needles. Figure S12. Dynamic light scattering results. Table S1. B-factors for CEF species observed in the shard crystals at different time delays. (PDF 1646 kb) [file 12915_2018_524_MOESM1_ESM.pdf]

Additional file 1.

Supplementary Figures S1-S12 and Table S1 for "Enzyme Intermediates Captured on-the-fly by Mix-and-Inject Serial Crystallography"

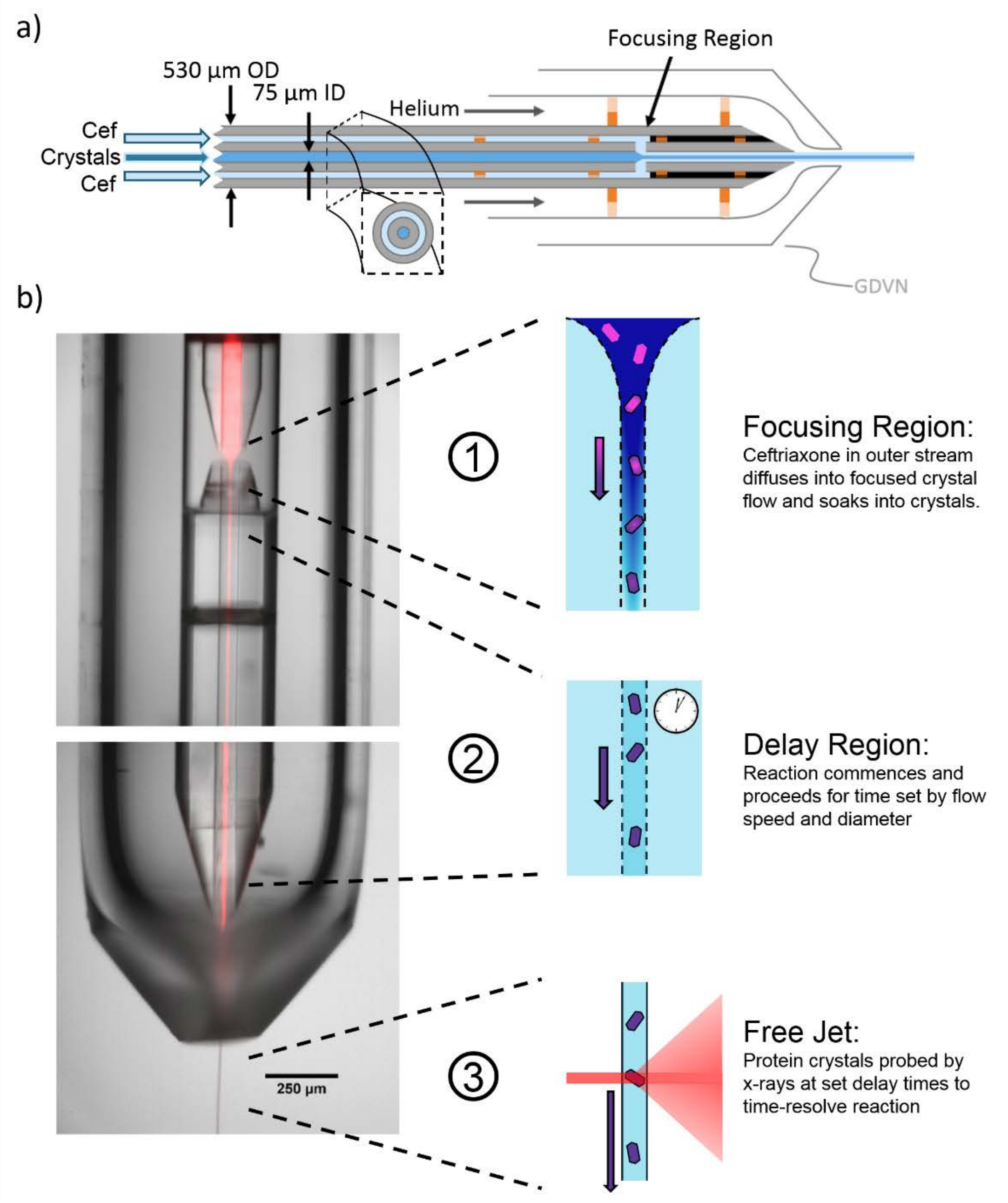

Figure S1. a) Schematic of short timepoint mixing injector. Capillary dimensions vary by timepoint. b) Composite image of fluorescent dye flowing through the sample capillary and water flowing through the buffer capillary. Cartoons illustrate the operating principle of each region of the device.

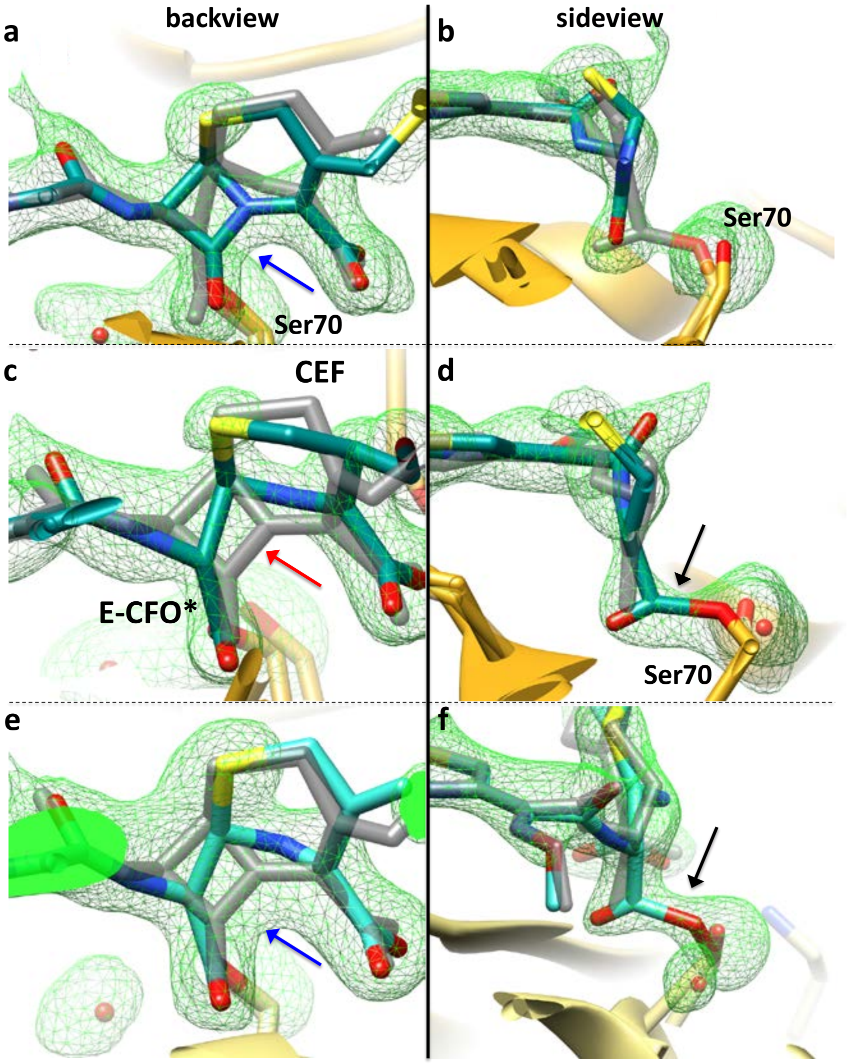

Figure S2. Selected views on the CEF binding site in the BlaC shard crystal form at various time delays. mFo-DFc SA omit electron density (green) contoured at 2.5σ. The first column shows the view on β–lactam ring from the backside in relation to Fig. 2 in the main text. The second column shows the side view to demonstrate cleavage of the lactam ring, and the covalent bond formation to Ser-70. The electron density is interpreted with two species (major species in blue, minor species in gray). **(a, b)** Electron density at 100ms in the BlaC shard crystal form, subunit B. The non-covalently bound, full length CEF is the main species (60%). The closed, uncleaved β-lactam ring nicely fits the electron density (a, blue arrow). The electron density between SER 70 and the open lactam ring is weak (b). The concentration of the covalently bound acyl adduct (E-CFO*) is low (40%). **(c, d)** Electron density at 500ms in the BlaC shard crystal form, subunit B. A covalently bound species (E-CFO*), where the β-lactam ring is opened, and the leaving group is split off, is the main species (blue, 70%). **(c)** The closed β-lactam ring poorly fits the electron density (red arrow), and the electron density is interpreted by an open lactam

ring (c). Strong electron density between SER 70 and the carboxyl of cleaved lactam ring indicates a covalent bond (d, black arrow). **(e, f)** Electron density at 500ms in the BlaC shard crystal form, subunit D. The full length CEF, and the E-CFO* acyl adduct are present approximately at equal proportions (50/50). The β-lactam ring fits nicely in the electron density (blue arrow), which can be interpreted by an uncleaved, full length CEF structure (e). However, in (f), strong electron density between the SER 70 and the cleaved open lactam ring (black arrow) indicates mixing-in of a covalently bound E-CFO* species.

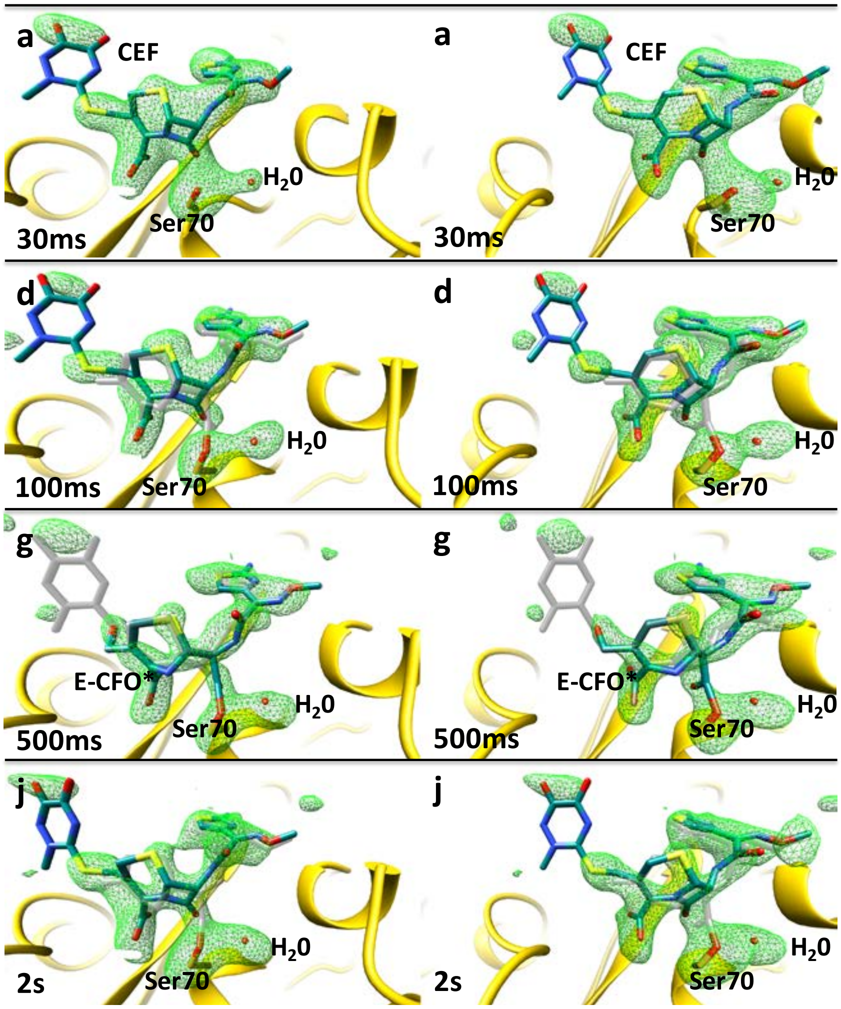

Figure S3. Simulated annealing omit maps, shard crystal form, subunit B (stereo representation) of the BlaC reaction with ceftriaxone, from 30 ms to 2 s. Panels are labeled with the same letters as in Fig. 2 (main text). Green: SA-omit difference density (2.5 σ contour). Blue: ligand main structural component, gray: minor structural component.

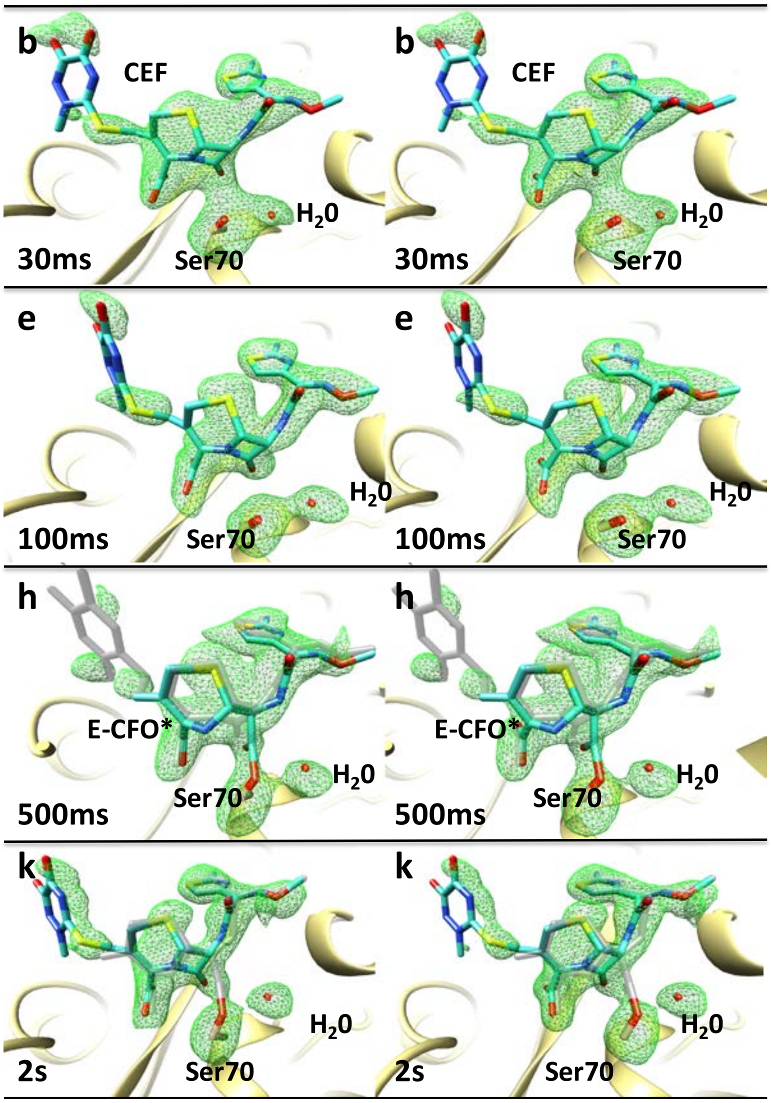

Figure S4. Simulated annealing omit maps, shard crystal form, subunit D (stereo representation). Images of the BlaC reaction with ceftriaxone, from 30 ms to 2 s. Panels are labeled with the same letters as in Fig. 2 (main text). Green: SA-omit difference density on the 2.5 σ contour level. Blue: ligand main structural component, gray: minor structural component.

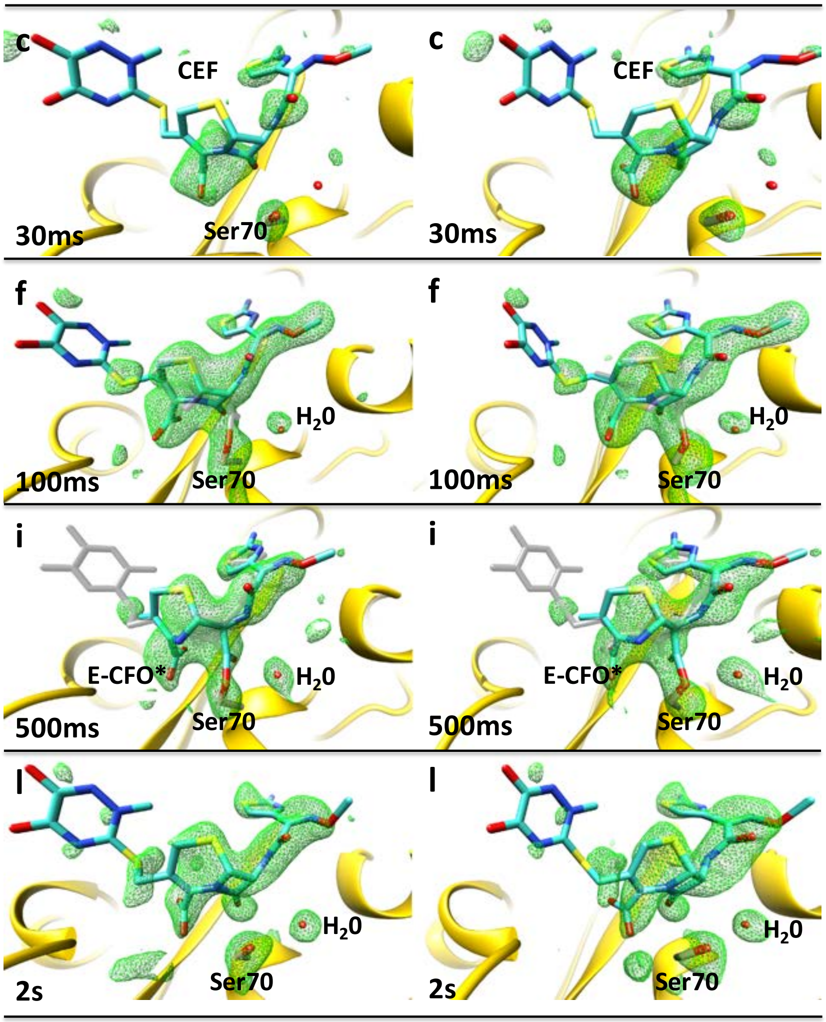

Figure S5. Simulated annealing omit maps, needle crystal form (stereo representation). Images of the BlaC reaction with ceftriaxone, from 30 ms to 2 s. Panels are labeled with the same letters as in Fig. 2 (main text). Green: SA-omit difference density, 2.5 σ contour level. Blue: main ligand component, gray: minor ligand component. The Ser-70 and the nearby water are marked.

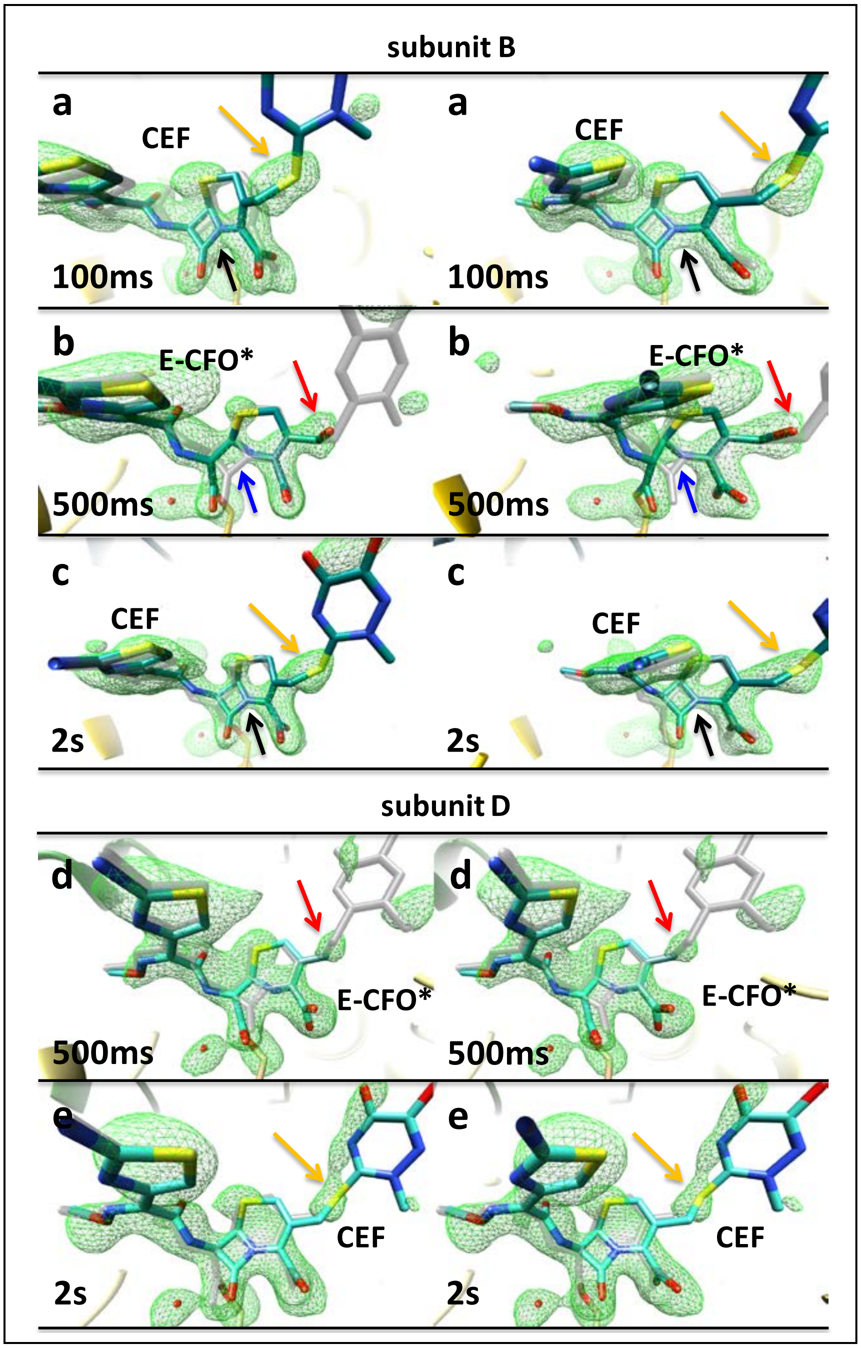

Figure S6. Backside view (stereo representation) of ceftriaxone binding to the catalytic cleft of BlaC, subunit B (a,b,c) and D (d,e) of the BlaC shard crystal form at various time delays, mFo-DFc SA omit electron density (green) contoured at 2.5 σ. The electron density is interpreted by different ceftriaxone species: main species in blue and minor species in gray. **(a)** Electron density at 100 ms (subunit B). Presence of prominent electron density for sulfur (orange arrow) and lactam ring nicely fits the electron density (black arrow) **(b)** Electron density at 500 ms (subunit B). Lactam ring is open (blue arrow), the absence of electron density for the sulfur (red arrow) is interpreted as the detachment

of R group, followed by the formation of an alcohol. **(c)** Electron density at 2 s (subunit B). Presence of prominent electron density for sulfur (orange arrow) and the lactam ring nicely fits the electron density (black arrow). The electron density is interpreted with a full length CEF structure. **(d)** Electron density at 500 ms (subunit D). Absence of electron density for the sulfur (red arrow) is interpreted as the detachment of R group. **(e)** Electron density at 2 s (subunit D). Reappearance of prominent electron density for the sulfur (orange arrow) and for the dioxo-triazine ring.

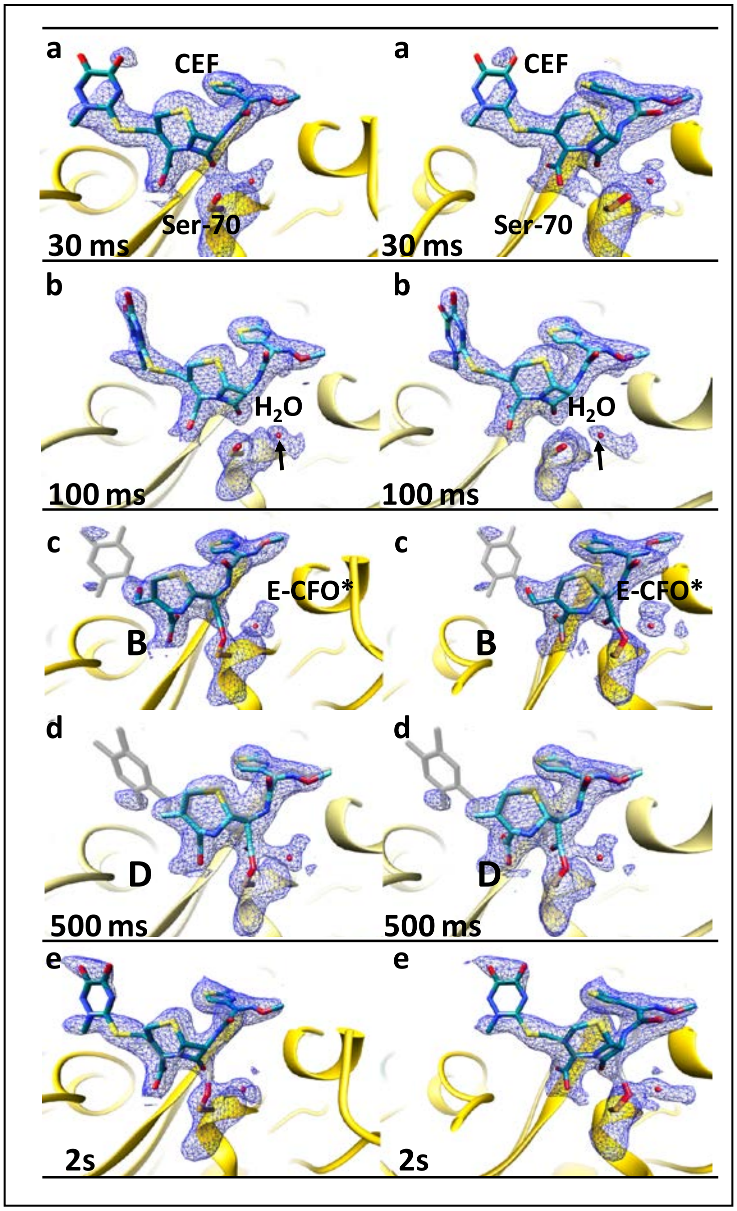

Figure S7. 2mFo-DFc electron density (blue, contour 1.1 σ, stereo representation) in the catalytic clefts of subunit B (a,c,e), and subunit D (b,d) of BlaC shard crystal form at different time delay after mixing. The main species is displayed in blue and the minor species in gray. (a) ES complex with the full length CEF non-covalently attached to the active site, (b) mixture of CEF and the covalently bound E-CFO* at 100 ms, (c) and (d) the covalently bound E-CFO* is the main component, (2s) reappearance of the full length CEF with a minor contribution of E-CFO*.

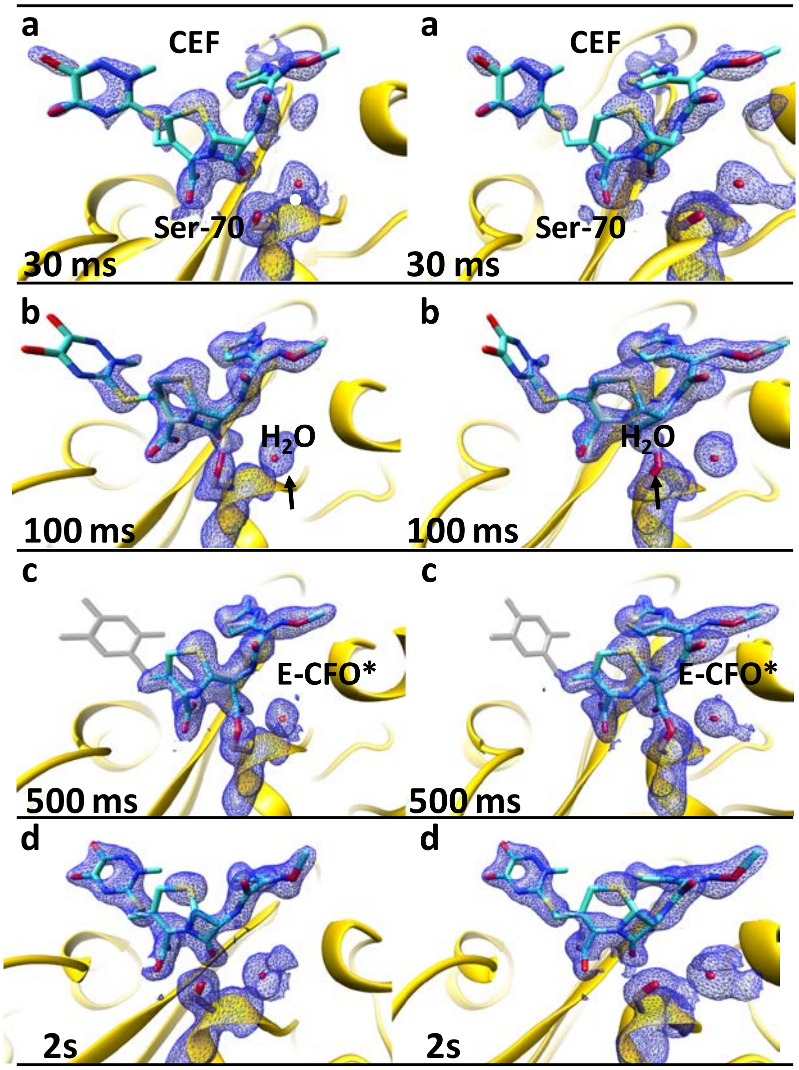

Figure S8. 2mFo-DFc electron density (blue, contour 1.1 σ, stereo representation) in the catalytic cleft of the BlaC needle crystal form at various time delays. The maps were calculated using extrapolated structures factors F_o^{ext} (see text) with N=9 for 30 ms and 100 ms delays, N=6 for 500 ms and N=5 for the 2 s delays. The electron density is interpreted by various ceftriaxone species. The main species is displayed in blue and the minor species in gray. **(a)** Formation of ES complex at 30 ms. The full length CEF model (blue) is displayed in the active site. **(b)** Early phase of the formation of a covalently bound E-CFO* adduct observed at 100 ms. The full length CEF model (blue) is displayed together with the minor E-CFO* species (gray), where the β-lactam ring is open and attached to Ser-70. **(c)** Fully cleaved and covalently bound adduct (E-CFO* in blue) at 500 ms. A small admixture of full length CEF (gray) is present. **(d)** 2s, steady state, CEF is dominant species as at 30 ms.

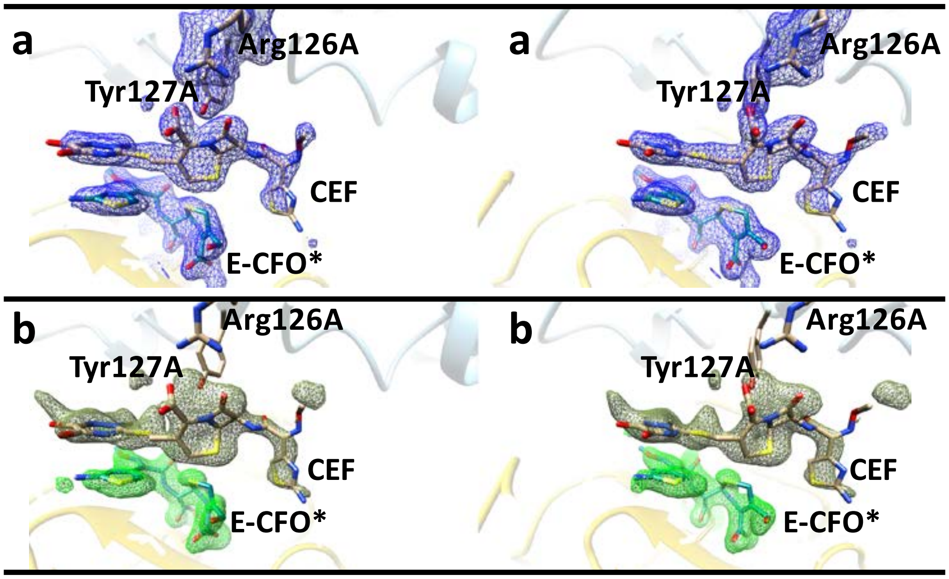

Figure S9. Details in the catalytic cleft of subunit B at 500 ms including the stacked molecule, which interacts with the adjacent subunit A (stereo representation). Details in subunit D with adjacent subunit C are similar. E-CFO*: covalently bound acyl intermediate, CEF: stacked full length ceftriaxone. (a) 2mFo-DFc electron density (1.1 σ contour level). (b) SA-omit maps (2.5 σ contour level for E-CFO*, and 2 σ for the CEF region).

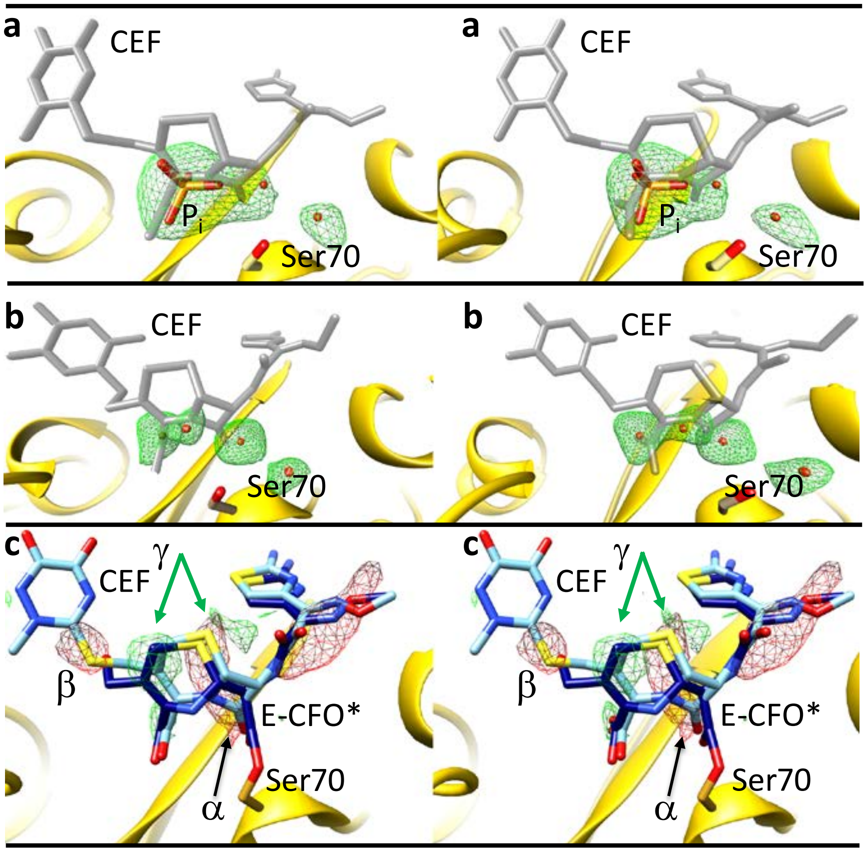

Figure S10. The catalytic cleft of BlaC. **(a)** Unmixed, ligand free structure of the BlaC shard crystals grown in phosphate buffer (stereo representation). The phosphate (P_i) is marked. The CEF ligand as found at several time delays after mixing is displayed in gray as a guide to the eye. Green electron density: mFo-DFc simulated annealing omit map (2.5 σ contour level) where all small molecules (water and phosphate) in the catalytic cleft were removed. **(b)** Unmixed, ligand free structure of the BlaC needle crystals grown in PEG (stereo representation). The CEF ligand as found at several time delays after mixing is displayed in gray as a reference. Green electron density: mFo-DFc simulated annealing omit map (2.5 σ contour level) where all waters in the catalytic cleft were removed. **(c)** Stereo representation of the F_o(500 ms)-F_o(100 ms) difference electron density map for subunit B of the shard crystals (contour levels: red -2.5 σ, green 2.5 σ). The full length CEF model which is the major species at 100 ms and the covalently bound acyl adduct (E-CFO*) which is the major species at 500 ms are shown in light and dark blue, respectively. The negative feature α (black arrow) is located on the lactam ring carbonyl. This shows that at 500 ms the carbonyl oxygen is displaced, the lactam ring is open, and the covalent adduct has formed. The negative feature β points to a higher sulfur occupancy at 100 ms compared to the 500 ms delay. This is evidence that the leaving group (R) detaches after 100 ms. The positive and negative density pairs on the

dihydrothiazine rings (indicated by 2 green arrows) show the shift of the ring positions from back (at 100 ms) to front (at 500 ms) after the lactam ring is opened.

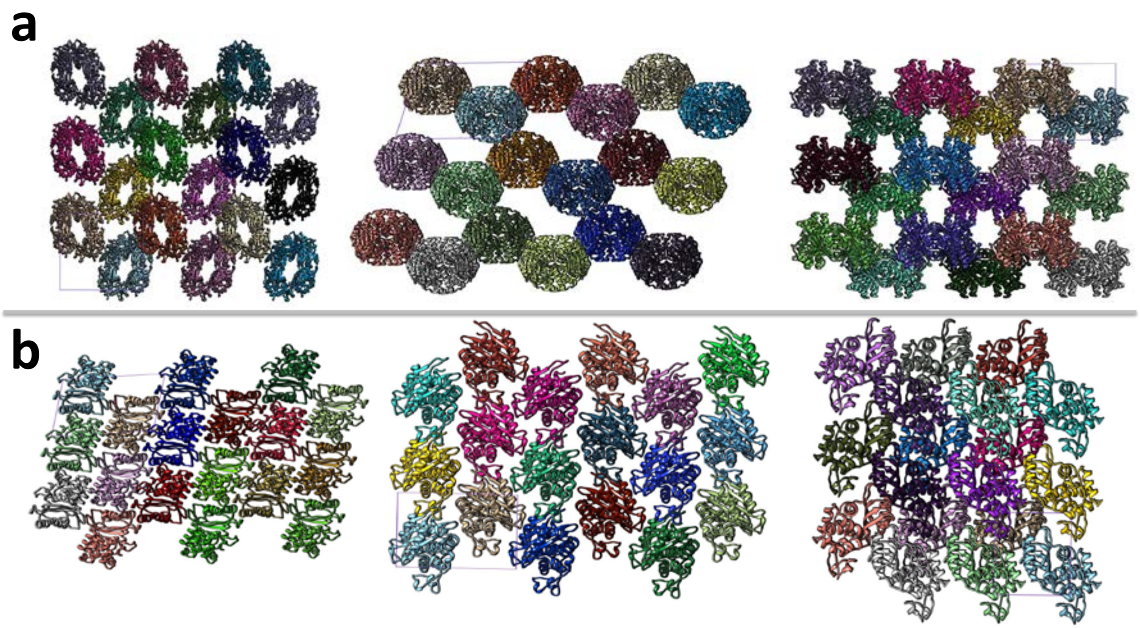

Figure S11. Crystal packing of BlaC in different crystal forms viewed from three different directions normal to the unit cell surfaces. 27 unit cells (three each in the directions along the unit cell axes) are displayed and viewed in orthographic projection. One of the unit cells is outlined for each respective view with faint purple lines. The unit cell volume of the shard crystal form is on the order of 805,000 Å^3 with 8 subunits in the unit cell (four molecules/asymmetric unit). The concentration of BlaC subunits is 16 mmol/L. The unit cell volume of the needles is about 110,600 Å^3 with 2 monomers in the unit cell. The concentration of BlaC is 33 mmol/L. (a) Shards, displaying large solvent channels in all 3 directions. (b) Needles, solvent channels are substantially smaller. Note, the display is not to scale. BlaC monomers in (b) appear larger than BlaC subunits in (a).

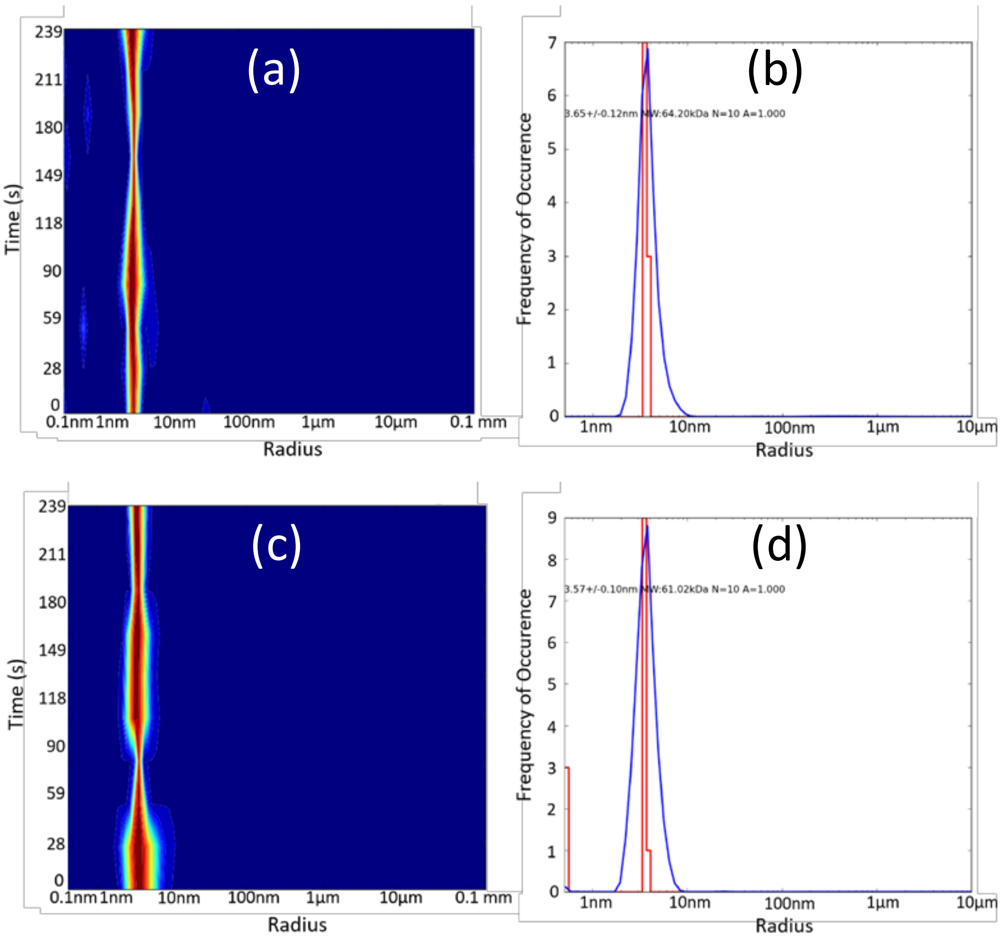

Figure S12. Dynamic Light Scattering on BlaC at 40 mg/mL at pH 5 using a DynaPro NanoStar M3300 (WYATT TECHNOLOGY). A 120 mW laser of 660 nm was used as the light source. For each measurement, the number of acquisitions was 10 and each acquisition time was 20 s. All measurements were carried out at 20 °C. (a) and (b) in 100 mmol/L Na-acetate buffer, (c) and (d) in 100 mmol/L Na-phosphate buffer. (a) and (c) show size distribution over time. (b) and (d) show the radius distribution. A very monodisperse species is present. From (b) and (d) accurate molecular weights can be calculated: (a) 64.2 kDa, (b) 61.0 kDa. The mass of a BlaC momomer is 30.6 kDa (*1*). The BlaC exists as a dimer at this pH in both buffers. Essentially the same result is obtained with 20 mg/mL BlaC.

Table S1. Average B-values for the various ceftriaxone species in the shard crystal form obtained with various refinement strategies: (i) *Refined with CEF only*: the full length CEF is inserted and refined. B-factors are separately listed for the active site full CEF and the leaving group. (ii) *Refined with CEF and E-CFO**: a mixture of CEF and E-CFO* is refined at 100 ms, 500 ms, and 2 s. For CEF, B-factors of the leaving group only are shown. *Stacked CEF* :The B-factors of the stacked CEF are given in the last line.

30ms

	B-subunit [Å^2]	*D-subunit [Å^2]*
Refined with CEF only		
Full CEF	69.95	68.36
Leaving group	94.85	98.81
Stacked CEF	100.96	102.38

100ms

Refined with CEF only		
Full CEF	51.68	54.59
Leaving group	84.92	78.86
*Refined with CEF and E-CFO**		
Leaving group	76.94	NA
E-CFO*	54.11	NA
Stacked CEF	70.48	73.30

500ms

Refined with CEF only		
Full CEF	59.03	53.96
Leaving group only	77.31	82.80
*Refined with CEF and E-CFO**		
Leaving group	60.89	66.88
E-CFO*	49.60	46.16
Stacked CEF	66.05	67.70

2sec

Refined with CEF only		
Full CEF	47.64	44.37
Leaving group	64.49	64.67
*Refined with CEF and E-CFO**		
Leaving group	64.96	61.65
E-CFO*	52.58	46.33
Stacked CEF	61.14	62.83
